# Maternal Vitamin B12 Status During Pregnancy and Its Association With Outcomes of Pregnancy and Health of the Offspring: A Systematic Review and Implications for Policy in India

**DOI:** 10.3389/fendo.2021.619176

**Published:** 2021-04-12

**Authors:** Rishikesh V. Behere, Anagha S. Deshmukh, Suhas Otiv, Mohan D. Gupte, Chittaranjan S. Yajnik

**Affiliations:** ^1^ Diabetes Unit, KEM Hospital Research Center, Pune, India; ^2^ Department of Clinical Psychology, Manipal College of Health Professions, Manipal Academy of Higher Education, Manipal, India; ^3^ Department of Obstetrics and Gynecology, King Edward Memorial (KEM) Hospital, Pune, India; ^4^ ICMR – National Institute of Epidemiology, Chennai, India

**Keywords:** vitamin B12, folate, pregnancy outcomes, offspring health, public health policy

## Abstract

**Background:**

Vitamins B12 and folate participate in the one-carbon metabolism cycle and hence regulate fetal growth. Though vitamin B12 deficiency is widely prevalent, the current public health policy in India is to supplement only iron and folic acid for the prevention of anaemia. Prompted by our research findings of the importance of maternal vitamin B12 status for a healthy pregnancy, birth and offspring health outcomes, we evaluated available literature evidence using a systematic review approach, to inform policy.

**Methods:**

A systematic search was performed for relevant Indian studies in the MEDLINE/PubMed and IndMed databases. We selected studies reporting maternal vitamin B12 status (dietary intake or blood concentrations), and/or metabolic markers of vitamin B12 deficiency (homocysteine, methylmalonic acid) or haematological indices during pregnancy and their associations with outcomes of pregnancy, infancy or in later life. Intervention trials of vitamin B12 during pregnancy were also included. Quality of evidence was assessed on the Grading of Recommendations Assessment, Development, and Evaluation (GRADE) system. We followed the Preferred Reporting Items for Systematic Reviews and Meta-Analyses (PRISMA) statement.

**Results:**

Of the 635 articles identified, 46 studies met the inclusion criteria (cohort studies-26, case-control studies-13, RCT’s -7). There is a high prevalence of vitamin B12 deficiency in Indian women during pregnancy (40-70%) (3 studies). Observational studies support associations (adjusted for potential sociodemographic confounders, maternal body size, postnatal factors) of lower maternal B12, higher homocysteine or an imbalance between vitamin B12-folate status with a higher risk of NTDs (6 studies), pregnancy complications (recurrent pregnancy losses, gestational diabetes, pre-eclampsia) (9 studies), lower birth weight (10 studies) and adverse longer-term health outcomes in the offspring (cognitive functions, adiposity, insulin resistance) (11 studies). Vitamin B12 supplementation (7 RCT’s) in pregnancy showed a beneficial effect on offspring neurocognitive development and an effect on birth weight was inconclusive. There is a high quality evidence to support the role of low maternal vitamin B12 in higher risk for NTD and low birth weight and moderate-quality evidence for higher risk of gestational diabetes and later life adverse health outcomes (cognitive functions, risk for diabetes) in offspring.

**Conclusion:**

In the Indian population low maternal vitaminB12 status, is associated with adverse maternal and child health outcomes. The level of evidence supports adding vitamin B12 to existing nutritional programs in India for extended benefits on outcomes in pregnancy and offspring health besides control of anaemia.

**Systematic Review Registration:**

[website], identifier [registration number]

## Introduction

Maternal undernutrition predisposes to a range of bad pregnancy outcomes including early pregnancy loss, congenital anomalies, poor fetal growth and perinatal morbidity and mortality (1). Poor fetal growth predisposes to long term ill-health in the offspring including increased risk of non-communicable diseases (2–4). Improving maternal nutrition during pregnancy is an important focus of public health initiatives in India.

Protein calorie undernutrition continues to be a substantial problem in India. In addition, there is an increasing recognition of the importance of maternal micronutrients (iron, vitamin B12, folate, vitamin C, vitamin D, pyridoxine, choline) in fetal growth and development (5–7). Vitamin B12 and folate act as methyl donors in one-carbon metabolism which affects cell growth and differentiation by influencing DNA synthesis and epigenetic regulation. Therefore, they are important regulators of fetal growth (8, 9). Homocysteine is an important indicator of deranged one-carbon metabolism and is raised in folate and vitamin B12 deficiency. Methyl Malonic Acid (MMA) on the other hand, is thought to be a specific marker for vitamin B12 deficiency.

Traditionally vitamin B12 deficiency is equated to pernicious anaemia (a genetic -immunological condition of intrinsic factor deficiency leading to poor absorption). Pernicious anaemia is a relatively rare cause of vitamin B12 deficiency in India. The ultimate source of vitamin B12 in nature is microbes. Vitamin B12 enters the food chain when animals obtain vitamin B12 by eating microbes. Humans obtain vitamin B12 from animal-sourced foods (milk, eggs, fish, meat) and bacterial contamination of water and food. Indians have a large prevalence of subclinical vitamin B12 deficiency due to poor intake of animal origin foods, owing to the religious and cultural practice of vegetarianism (10). Poverty also contributes because non-vegetarian foods are expensive. Though vitamin B12 deficiency is widely prevalent, vitamin B12 supplementation is not a part of current public health policy in India. Only iron and folic acid supplements are provided to improve adolescent and maternal-child health. Folic acid supplements are also prescribed to prevent Neural Tube Defects (NTD).

Maternal vitamin B12 and folate have been reported to be associated with maternal and child health outcomes (11, 12). Two meta-analyses of studies (13, 14) report that maternal vitamin B12 deficiency/insufficiency is associated with an increased risk of low birth weight and preterm births. We have investigated the role of maternal vitamin B12 and folate nutrition, and derangement of one-carbon metabolism in pregnancy outcomes as well as the long-term outcomes in the offspring. Our studies include prospective birth cohorts and double-blind randomized controlled trials (15). Our findings have established a high prevalence of vitamin B12 deficiency in our population and its detrimental effects on maternal and fetal health. Studies in South India have also highlighted the role of vitamin B12, folate and iron in pregnancy anaemia and shown beneficial effects of combined supplementation. It is therefore important to investigate reports from other parts of India to help expand the evidence base.

We reviewed the literature for studies done in India examining the prevalence of vitamin B12 deficiency and its association with pregnancy and neonatal outcomes and long-term effects on the baby. We also reviewed evidence from supplementation studies of B12-folate rich foods or vitamin B12 supplementation (with or without multiple micronutrients - MMN), in influencing these outcomes.

## Method

### Search Criteria

A comprehensive literature search was performed in the PubMed and IndMed databases. A search strategy based on the following keywords and medical subject headings (MeSH) was used: “cobalamin,” “vitamin B12,” “vitamin B12 insufficiency,” “vitamin B12 deficiency,” “holotranscobalamin,” “homocysteine,”, “hyperhomocystenemia”, “methylmalonic acid”, “folate”, “anemia,”, “pernicious anemia”, “macrocytosis” “neural tube defects,”, “congenital anomalies”, “pre-eclampsia”, “eclampsia”, “pregnancy complications”, “birth weight”, “physical growth”, “development”, “cognition,” “cognition disorders, “cardiovascular diseases”, “metabolic syndrome”, “supplementation”, “pregnancy,” “pregnant women,” and “India.” Search words were combined by using Boolean operators (AND, OR). There was no restriction on the publication period in the search strategy.

### Data Extraction and Quality Assessment

The titles and abstracts were independently evaluated by two reviewers (RVB and AD) and the required information was extracted using a semi structured format. Any discrepancy in data extraction was resolved by consensus between the reviewers.

We included longitudinal and cross-sectional observational studies from India that reported maternal vitamin B12 status (dietary intake or blood concentrations of vitamin B12, holoTC), and/or metabolic markers of vitamin B12 deficiency (homocysteine, methylmalonic acid) or haematological indices. Intervention trials of vitamin B12 (with or without MMN) or B12-folate rich foods during pregnancy were also included. Both community and hospital-based studies were included. Only human studies were included. Studies that report effects of other maternal micronutrients (e.g., iron and folate) without vitamin B12 are outside the scope of this review. We included associations with folate when reported along with vitamin B12 or as B12 folate ratio. If the results of a study were reported in more than one publication, the study with the most complete information pertaining to our review outcomes was used. No attempt was made to obtain unpublished data. The main outcomes reported in the reviewed studies were identified.

Prevalence data was extracted and compiled from the included studies which reported vitamin B-12 or folate deficiency and hyperhomocysteinemia with clearly defined cut-offs in pregnant women at any trimester, including delivery. The main outcomes in pregnancy and offspring were categorized as: 1) Pregnancy outcomes: (pregnancy anemia with macrocytosis, pregnancy loss, pre-eclampsia, gestational diabetes mellitus, pre-term delivery); 2) Offspring outcomes at birth: a) congenital anomalies, b) birth size (anthropometry, Small for Gestational Age - SGA, Low Birth Weight - LBW); 3) Later life health outcomes in the offspring: a) offspring vitamin B12 status b) Physical growth and development, c) Cardiovascular, metabolic and endocrinal functioning, and d) cognitive functioning. We followed the Preferred Reporting Items for Systematic Reviews and Meta-Analyses (PRISMA) statement.

The quality of evidence for the main outcomes was assessed on the Grading of Recommendations Assessment, Development, and Evaluation (GRADE) system of rating quality of evidence (16). GRADE provides a systematic approach for evaluation of a body of evidence. GRADE specifies four categories - high, moderate, low, and very low which reflects the level of confidence on the estimates of the effects. GRADE ratings were performed by two reviewers independently (RVB and AD) and an average was taken.

### Statistical Considerations

The observational studies were evaluated for the strength of associations. We reported standardized beta coefficients or adjusted odds ratios where available for association studies. Studies were evaluated on parameters such as type of study, sample size estimation, adjustment for relevant confounders. We also examined if the results support causal associations (Mendelian randomization, associations with genetic polymorphisms, intervention trials).

## Results and Comments

**Figure 1:**
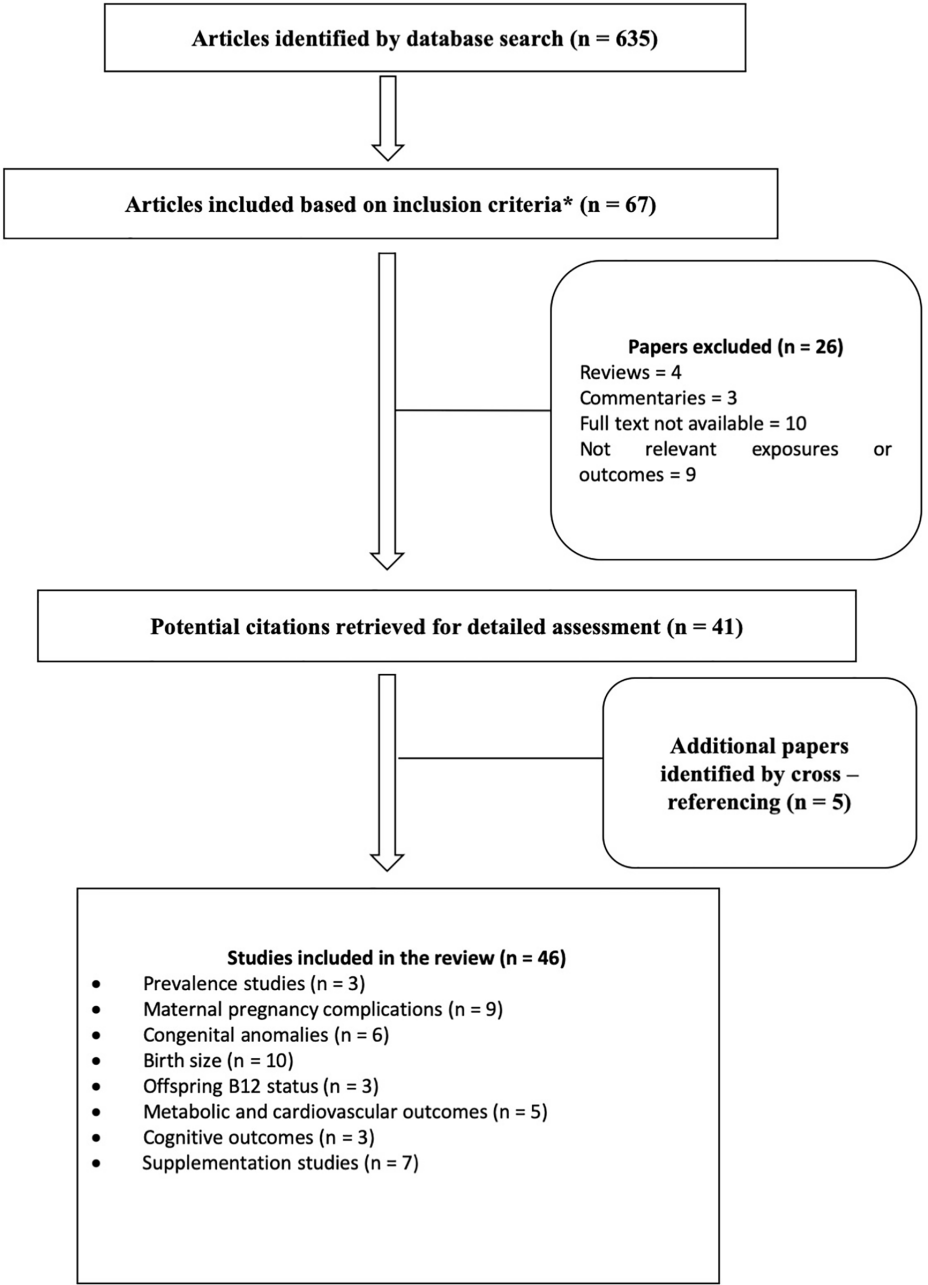
.Flow diagram illustrating selection of articles in review.

From our search 635 articles were identified (**Figure 1**) and after evaluation of title and abstracts 67 articles fulfilling our inclusion criteria were identified, 26 studies were excluded (reviews, commentaries, non-availability of full texts and irrelevant exposures or outcomes). Another 5 articles were identified by lateral search and cross referencing, making a total of 46 studies for final evaluation. The results of prevalence studies are reported in **Table 1** and results of outcome studies with their GRADE rating for quality of evidence are summarized in **Table 2**. A detailed description of reviewed studies is provided as **Supplementary Tables 1–9.**

**Table 1:** Indian studies on prevalence of vitamin B12, folate deficiency, or hyperhomocysteinemia during pregnancy.

Sl. No.	Title/Author	Region	Time point	Findings
1	Iron, folate, and vitamin B12 stores among pregnant women in a rural area of Haryana State, India Pathak et al. (17)	Haryana (rural – North India)	≥28 week pregnancy	74.1%, 67.7%, 26.3%, of the women had low vitamin B12 (<200pg/ml~150pmol/L), ferritin (<12ng/ml), and folate (<3ng/ml), respectively. Concomitant deficiencies of iron, folate, and vitamin B12 occurred in 16.2% of the women
2	Low plasma vitamin B12 in pregnancy is associated with gestational ‘diabesity’ and later diabetes Krishnaveni et al. (18)	Mysore (Urban low socioeconomic status women – South India)	30 weeks gestation	In 774 women in third trimester, 43% had low B12 (<150pmol/L) and 4% had folate deficiency. Low B12 prevalence was 50.7% in Hindu mothers (predominantly vegetarian) and 35.6% in Muslim mothers (non-vegetarian).
3	Vitamin B12 and folate concentrations during pregnancy and insulin resistance in the offspring: the Pune Maternal Nutrition Study Yajnik et al. (19)	Pune (rural – West India)	18 and 28 week pregnancy	In ~600 women 60% had low B12 (<150 pmol/L), at 18 weeks with median concentrations of 135pmol/L. At 28 weeks 70% of mothers had low vitamin B12 (<150 pmol/l) median concentrations 122pmol/L, 90% had high MMA (>0.26 μmol/l) and 30% had raised tHcy concentrations (>10 μmol/l) Folate deficiency was seen in 1% women.
4	Vitamin B12 and folic acid supplementation and plasma total homocysteine concentrations in pregnant Indian women with low B12 and high folate status Katre et al. (20)	Pune (rural and urban – West India)	17 weeks pregnancy	In 200 pregnant women, B12 deficiency (<150pmol/L) seen in 80% rural and 64% urban women. Hyperhomocystenenia (>10 μmol/l) seen in 28% rural and 26% urban women
5&6	Vitamin B12 intake and status in early pregnancy among urban South Indian women Finkelstein et al. (21), Samuel et al. (22)	Bengaluru (urban low socioeconomic status women - South India)	First measurement at ≤14 weeks of gestation and at 2^nd^ and 3^rd^ trimester	In 366 pregnant women low vitamin B 12 concentration (<150pmol/L) was observed in 51.1% of the women, Elevated MMA (>0.26 µmol/l), elevated homocysteine (>10 µmol/l) and low erythrocyte folate (<283 nmol/l) were observed among 75.8, 43.3 and 22.2% of the women, respectively. In a subset of 77 women median plasma B12 concentrations was 150pmol/L in 1^st^ and 2^nd^ trimesters and fell to 139pmol/L in third trimester.
7	Imbalance of folic acid and vitamin B12 is associated with birth outcome: an Indian pregnant women study Gadgil et al. (23)	Pune (Hospital based sample – West India)	36 weeks of gestation	In 50 women coming for antenatal care, Vitamin B12 concentration was <150 pg/ml (~110pmol/L) in 35% women. 82% women showed a high folic acid concentration (above 3–12 ng/ml) Total homocysteine concentration was above 9.5 μmol/l in 39%.
8	A prospective study of maternal fatty acids, micronutrients and homocysteine and their association with birth outcome Wadhwani et al. (24)	Pune (Hospital based sample – West India)	1^st^ measurement at 16-20 week pregnancy and subsequently at 28-30 weeks and at the time of labor.	In 109 women, plasma vitamin B12 level (<150 pg/ml) was 22.22%, 31.64% and 42.04% at the three time points. Plasma folate levels (<10 ng/mL) were 46.66%, 56.25% and 61.79% respectively Plasma homocysteine levels (>10 nmol L) were 7.77%, 7.5% and 22.47% respectively.

**Table 2:** Table summarizing the outcomes reviewed with GRADE rating for quality of evidence.

Reviewed studies		Observations	Comments	GRADE Rating(Average score)*	Level of evidence
**Prevalence of B12 deficiency** **in pregnancy**		B12 deficiency: 50-70% (17–21, 23, 24). Raised Methylmalonic acid: 70-90% (19, 22). Hyperhomocysteinemia: 28-43% (19, 20, 22). B12 measurements were lower in the third trimester of pregnancy as compared to early pregnancy.	Prevalence reported from both community and hospital based samples in rural and urban populations. Standardized assay techniques used to measure B12 concentrations with similar cut off values across studies. Estimates of prevalence rates are reliable	**—**	—
**Pregnancy** **outcomes**	**Anemia with macrocytosis**	Two cross-sectional studies (25, 26) reported 40-50% prevalence of macrocytosis in pregnant women with anemia	Small sample size	1.5	Low
	**Recurrent pregnancy losses**	Two case control studies Higher homocysteine (2 studies) (27, 28), low B12 (1 study) (27), low B12-folate ratio (1 study) (28) in women with RPL	These Case control studies have methodological limitations (inadequate control for confounding, sample selection and matching not adequately described)	2.5	Low
	**Higher risk for pre-eclampsia**	Two case control studies. Higher maternal homocysteine (2 studies) (29, 30) and low B12 (1 study) (30) in preeclamptic women		2.0	Low
	**Higher risk for Preterm delivery**	2 case control studies. Higher homocysteine and higher B12 levels in mothers delivering preterm due to preeclampsia) (31, 32)		2	Low
	**Higher risk for GDM**	One observational study (18) in a prospective birth cohort. Higher risk for gestational diabetes in B12 deficient women. One RCT (33) supplementation with micronutrient snack reduced incidence of GDM by 50%	Observations adjusted for confounding. Supported for causality by intervention trial (micronutrient snack of green leafy vegetables and milk powder)	3	Moderate
**Congenital** **anomalies**	**Higher risk for NTD in offspring**	Three singe center case control studies (34–36) and one multicenter study (37) Higher homocysteine in mothers (2 studies) (36, 37), fathers and neonates (1 study) (34), vegetarian diet in mothers (1 study) (35), low maternal b12 status (1 study) (36) and low maternal transcobalamin levels (1 study) (37) was associated with higher risk for NTD. Maternal 776C>G polymorphism in TCN2 was strongly predictive of NTD in the offspring (1 study) (37)	A large multicenter case control study ~ 700 subjects, NTD detected by fetal ultrasound, maternal blood concentrations measured at time of detection confirmed association of risk of NTD with maternal transcobalamin levels. Supported for causality by associations with genetic polymorphisms of B12 metabolism.	4	High
	**Increased risk for congenital heart defects**	1 case control study (38) Higher maternal homocysteine in cases with congenital heart defects in offspring	Maternal concentrations measured 9-15 months after delivery (no temporal correlation between exposure and outcome. Small sample size	1.5	Very low
	**Risk for orofacial clefts**	One case control study. Exclusive vegetarianism in mothers associated with higher risk of orofacial clefts in mother (39)	Vegetarian diet is a proxy for B12 status. Maternal concentrations or dietary intakes not assessed	1	Very low
**Birth** **outcomes**	**Higher Neonatal morbidity**	One case control study reported higher neonatal morbidity in anemic pregnant women with macrocytosis (26). In one intervention trial (40) micronutrient supplementation (inclusive of B12) in undernourished mothers reduced neonatal morbidity	Small sample size in case control study. Intervention trial performed in a selected sample of undernourished mothers. Supplementation given in late pregnancy.	2.5	Low
	**Higher risk** **for LBW**	Lower consumption of GLV and fruits (1study) (41), lower maternal B12 status (2 studies) (24, 42), lower maternal B12/folate ratio (1 study) (23) and lower cord B12 status (1 study) (43) was associated with lower birth weight. In three studies (24, 42, 44) higher maternal homocysteine was associated with birth weight additionally supported for causal association by Mendelian randomization technique (1 study) (44). A micronutrient food based intervention did not improve birth weight or fetal growth (2 studies) (45, 46). One RCT with 50mcg B12 starting from early pregnancy did not improve birth weight (47). Micronutrient supplementation (inclusive of B12) in late pregnancy in undernourished mothers reduced incidence of LBW (40).	Many observational studies with adjustments for confounding. Mendelian randomization technique supports causal association of high maternal homocysteine (marker of B12 deficiency) with LBW. Inference of causality from RCT is inconclusive.	4	High
	**Higher risk for IUGR, SGA**	Lower maternal B12 status associated with higher risk of IUGR (1 study) (48). In a case control study nested within a birth cohort SGA group had higher maternal homocysteine during pregnancy (1 study) (49). Women with lowest dietary intakes of B12 rich foods and higher intake of folic acid supplements during pregnancy had highest risk for delivering SGA babies (1 study) (50)	Case control study with matched sample and adjusted for confounders. Systematic assessment of dietary intakes.	3	Moderate
	**Other neonatal size (smaller length, head circumference)**	Higher consumption of GLV and milk by mother was associated with higher birth length, head, chest circumference (1 study) (41). Higher maternal folate to B12 ratio was associated with birth length chest circumference and head circumference (1 study) (23).	GLV and milk products are a proxy measure of B12 intake. Small sample size of observational study that examined folate/B12 ratio	2.0	Low
**Later** **life outcomes**	**Lower vitamin B12 status in offspring**	Maternal B12 status in pregnancy associated with B12 status in offspring at 6 weeks (1 study) (21), 2 years (1 study) (51) and 9 years of age (52)		3	Moderate
	**Offspring metabolic outcomes (adiposity, insulin resistance, bone mineral density)**	Higher dietary intake of GLV milk products during pregnancy associated with higher bone mineral density in childhood but no associations with B12 concentrations (1 study) (53). Low maternal B12 status and low B12-high maternal folate pattern was associated with higher insulin resistance in offspring at 6 years (1 study) (19) while maternal B12 showed no association at 9 years in another study (54). High maternal folate associated with higher adiposity and insulin resistance in childhood (2 studies) (19, 54). High homocysteine associated with higher glucose concentration in child (1 study) (54).	Observations in cohort studies adjusted for confounding	3	Moderate
	**Cardiovascular outcomes (Offspring stress response and HRV)**	Higher maternal B12 deficiency and higher homocysteine in pregnancy associated with greater cortisol and heart response to stress in childhood (1 study) (55). Low maternal B12 status in pregnancy associated with reduction in HRV in child (56)	Observations in prospective cohorts with adjustment for cofounders but outcomes examined in smaller subset of participants	2.5	Low
	**Offspring cognitive functions**	Low maternal B12 status associated with poorer cognitive function in offspring at 2 years and 9 years (2 studies) (57, 58) and there was no association with B12 in one study (59). Higher maternal folate associated with better motor development at 2 years (57) and better cognitive functions at 9 years (59). One RCT showed supplementation with 50mcg B12 starting from early pregnancy improved language functions at 30 months (60) but not 9 months (61). Higher maternal homocysteine was associated with poorer cognitive outcomes (60, 61)	Supported for causality by observations from RCT	3.5	Moderate

GRADE ratings (1 – Very low, 2- Low, 3 – Moderate, 4 – High).

*Average of ratings by 2 reviewers.

### Prevalence of B12, Folate Deficiency, Hyperhomocysteinemia During Pregnancy

Three studies (17, 20, 21) reported only prevalence estimates of deficiency of B12, folate and raised homocysteine in pregnancy. Five others also reported pregnancy and/or offspring health outcomes, in addition to prevalence. In these studies, plasma concentrations of B12 less than 150 pmol/L (or 200pg/ml) is considered as deficiency.

Geographical variations in prevalence of vitamin B12 deficiency in pregnancy were observed. Highest prevalence is reported from community studies in Western and Northern parts of India (70-74%) (17, 19, 20). Estimates from Southern India (Karnataka) are lower at 51% (18, 21, 22). Prevalence rates for eastern and north-eastern regions and other states of India have not been reported. Concentrations of vitamin B12 from early pregnancy (14- 20 weeks) to later pregnancy (28 weeks to delivery) show a fall in vitamin B12 concentrations by 10-15pM and a rise in prevalence of vitamin B12 deficiency by 10-20% (19, 21, 24).

The prevalence rates of inadequacy of dietary intake of vitamin B12 (<1.2mcg/d) as assessed on the food frequency questionnaire across the three trimesters of pregnancy from south India were reported as 25%, 11% and 10% respectively (50). Rates for folate deficiency are reported to be 20% at a cut off value of plasma folate <3ng/ml (17) and 40% at plasma folate <10ng/ml) (24). These prevalence rates of folate deficiency are much lower than the observed prevalence rates for vitamin B12 deficiency.

Levels of homocysteine are raised in both vitamin B12 and folate deficiency (Homocysteine greater than 10 µmol/L is considered as hyperhomocysteinemia in pregnancy). Homocysteine levels are lower in pregnancy as compared to a non-pregnant state; levels increase with the duration of gestation. Prevalence rate of hyperhomocysteinemia reported in pregnancy is 28 – 43% (19, 20, 22). Methlymalonic Acid (MMA) is raised in vitamin B12 deficiency only. Prevalence rates of Elevated MMA (>0.26 μmol/L) was reported in 76-90% (19, 22).

### Comment

These observations are similar to results of a world-wide meta-analysis of fifty-seven studies which reported higher prevalence from the Indian cohorts (43 – 74.1%) (13). Comparison of vitamin B12 deficiency rates from other countries in the Indian subcontinent show a rate of 49% in Nepal (B12<150pM) (62) and 56% in Bangladesh (B12 < 185pM) (63). The worldwide pooled trimester wise estimates of prevalence of B12 deficiency were 21%, 19%, 29% in the first, second and third trimester respectively and the rates for folate deficiency during pregnancy in India are comparable to worldwide estimates (13). The public health policy of iron and folic acid supplementation during adolescence and pregnancy may contribute to Indian women being fairly folate replete during pregnancy. A study on 979 women of reproductive age (15-35 years) from a rural district in Telangana reported vitamin B12 deficiency (B12<203pg/ml) in 45% of the population, suggesting that many women at the time of conception are already vitamin B12 deficient (64).

Indian women have lower stores of vitamin B12 due to vegetarianism ascribable to both socio-cultural practices (religion) and lower economic status. Absorption studies in the Indian population have demonstrated adequate absorption in more than 85% of the population which has a very high prevalence of vitamin B12 deficiency (65). This suggests that the deficiency is due to poor dietary intake as opposed to the classical pernicious anaemia where there is a physiological defect in absorption.

Measurement across trimesters shows a consistent fall in vitamin B12 levels. This is ascribed to progressive hemodilution in pregnancy and also to reducing concentrations of binding protein (haptocorrin) and increased excretion in urine due to increased glomerular filtration rate. Transfer of nutrients from mother to fetus also contributes to lower maternal status during pregnancy (reverse causality). Holotranscobalamin (active form of vitamin B12) levels remain relatively stable in the latter half of pregnancy and are a more reliable indicator of vitamin B12 status in pregnancy (66, 67). However, the reviewed studies did not measure holotranscobalamin levels.

There is a high prevalence (50-70%) of B12 deficiency (<150pM) reported from Western and Southern parts of India. Vitamin B12 measurements were lower in the third trimester of pregnancy as compared to early pregnancy. The prevalence rates have been reported from both community and hospital based samples from rural and urban populations. Standardized assay techniques were used to measure vitamin B12 concentrations with similar cut off values across studies. The estimates of prevalence rates appear reliable. Given the wide cultural and geographical diversity of India, there is a need for national studies on representative populations to estimate prevalence rates in different regions.

### Maternal Pregnancy Complications

Nine observational studies reported maternal pregnancy complications, including pregnancy anaemia with macrocytosis (25, 26), recurrent pregnancy loss (27, 28), preeclampsia and preterm delivery (29–32), and Gestational Diabetes Mellitus (GDM) (18) (**Supplementary Tables 1**, **2**). One study reported gestational diabetes as an outcome in a supplementation trial (33) (**Supplementary Table 8**).

#### Observational Studies

Two cross-sectional studies (25, 26) reported a prevalence of macrocytosis of 40-50% in anaemia during pregnancy. One study reported higher neonatal morbidity in offspring born to mothers with macrocytosis (29.7% vs 13.4% in women with microcytic anaemia). The observations have been made on a small sample size. The GRADE rating for studies on macrocytosis in anaemia of pregnancy was very low. There are no studies which report serial measurements of haemoglobin and red cell indices during three trimesters. The adequate folate status and low iron status may mask B12 related macrocytosis in these women leading to under recognition of the problem.

Two studies reported recurrent pregnancy loss (RPL) as outcomes. Hyperhomocysteinemia (OR = 7.02, 95%CI 3.8,12.8) and vitamin B12 deficiency (OR=16.39, 95%CI 7.7, 34.8) were significant risk factors for recurrent pregnancy loss (27). The other study (28) additionally reported lower B12 to folate ratios (18.46 vs 124, p<0.001) in non-anaemic women with history of idiopathic recurrent pregnancy loss. Raised homocysteine was observed to be consistently associated with RPL. Two studies (28, 68) (**Supplementary Table 8**) report a predictive association of carrier status of MTHFR C677T polymorphisms with RPL (OR = 1.9). This polymorphism is known to be associated with impaired folate metabolism and elevated homocysteine levels. However, the study by Puri et al. (27), did not confirm the association with MTHFR polymorphisms.

Two studies (29, 30) compared vitamin B12, folate, homocysteine levels between 50 preeclamptic and normotensive pregnant women. Both studies found higher homocysteine levels in the preeclamptic group (16.4vs 8.19umol/L, p<0.001) and one study (30) additionally reported lower vitamin B12 levels (321 vs. 391 pg/ml, p<0.001) in cases. Two studies (31, 32) examined preeclampsia and preterm delivery as outcomes and reported higher B12 and homocysteine levels in these women compared to normotensive pregnant women. This rather counterintuitive finding maybe related to impaired renal function due to preeclampsia. Higher homocysteine was also associated with higher systolic and diastolic blood pressure (r=0.2, p<0.001).

One study in a prospective birth cohort (18) examined the association of vitamin B12 and folate concentrations (measured at 30 weeks gestation) with gestational diabesity (glucose tolerance test), adiposity (BMI, skin fold thickness), insulin resistance and later diabetes (follow up at 5 years). An oral glucose tolerance test (100gm) was performed and GDM was defined using the Carpenter-Coustan criteria. Vitamin B12 deficient women had higher incidence of gestational diabetes (OR – 2.1, 95%CI 1.1, 3.6) and greater adiposity, insulin resistance and diabetes prevalence 5 years after pregnancy. In the vitamin B12 deficient group the incidence of gestational diabetes increased with higher folate concentrations.

#### Intervention Studies

A pre-conceptional intervention trial (Mumbai Maternal Nutrition Project - MMNP) (45) with a micronutrient rich samosa (containing green leafy vegetables, fruits and milk powder) supports a possible role for supplementation with micronutrient rich food in reducing risk for GDM (33). Non-pregnant women (n=6513) were randomized to receive either a micronutrient rich or a ‘control’ samosa daily. Both groups received iron-folic acid supplements. Oral glucose tolerance test (75gm glucose) results were available in ~1000 pregnancies. GDM was defined based on the WHO 1999 criteria. There was a significant reduction in prevalence of GDM (from 12.4% to 7.3%) in the intervention group.

#### Comment

The GRADE evidence for RPL, preeclampsia and preterm delivery outcomes is of low quality. The case control studies suffer shortcomings in their design and analysis. Two studies did not report the time point of sample collection (29, 30). B12 concentrations fall from early to late pregnancy. There was inadequate control for confounders (31, 32).

The quality of evidence for gestational diabetes is moderate. This observation is reported from a cohort study with adequate controlling for confounders. However, the vitamin B12 GDM associations were cross-sectional. An RCT designed from findings of PMNS might support a role for micronutrient rich foods in reducing risk of GDM, though not specifically for B12 and folate. Food based intervention trials have certain inherent limitations: 1) They cannot be completely blinded; 2) The effects may not be ascribable to individual nutrients. The snack containing powdered green leafy vegetables, fruits and milk was presumed to contain folate and B12, however, measurements in a limited number did not show any improvement in circulating vitamin B12 levels with this intervention in the MMNP.

Available evidence suggests a possible role for low maternal B12 in increasing the risk for GDM, however reverse causality cannot be ruled out. Similarly, it is not possible to draw an inference on the risk of RPL and preeclampsia.

### Congenital Anomalies

Four case control studies (34–37) reported associations between Neural tube defects and folate, vitamin B12 and homocysteine concentrations in the mother. Two studies reported other congenital anomalies (heart defects, orofacial cleft) (38, 39) (**Supplementary Table 3**).

#### Observational Studies

The NTD studies are reported from North India (34–36) and one was a multicentre study (37). Higher maternal homocysteine was associated with higher risk for NTD (36) (14.6 vs. 4.4 umol/L p<0.001). One study also reported higher paternal homocysteine as an independent risk factor for NTD (34) (adjusted OR = 26.5, 95%CI 2.6, 262.4). High homocysteine levels were observed in NTD neonates (34) (17.9µmol/L vs 11.9µmol/L, p=0.02). Vegetarian diet in the mothers (OR=1.77, 95%CI 0.02, 3.05) (35) and lower maternal B12 levels (191.8 vs. 478 pg/ml, p=0.002) (36) were associated with higher NTD risk. These case control studies report maternal concentrations only after birth. However, neural tube closure occurs in early pregnancy and hence these observations do not provide an estimate of maternal concentrations during the neuro-developmental window period. One of the largest (318 cases/702 controls) multi-centric study of NTD’s was done in four centres in India (37). In this study, a temporal association between the plasma levels and NTD could be established as samples were collected at the time of diagnosis of NTD (by antenatal ultrasound). It identified low maternal transcobalamin (65.3% vs 55.2% in NTD versus controls, p=0.003) and high homocysteine to be associated with NTD. Causality was supported by predictive association of maternal TCN2 polymorphisms (Maternal 776C>G polymorphism in TCN2 was strongly predictive of NTD in the offspring).

Three studies reported maternal genetic polymorphisms (folate and vitamin B12 metabolism) (**Supplementary Table 9**) in relation to risk of NTD. The MTHFR C677T polymorphism increased risk for NTD in one study (69) (OR = 2.69) while another study reported an ethnic difference with Muslim mothers having a higher frequency of the TT genotype (8.7% vs 1.1% in Hindu mothers) which reflected in increased risk of NTD by 12.9 times compared to Hindus (35). The large multicentric study found no association with the MTHFR C677T polymorphism probably because the allele frequency was quite low, however other folate genetic determinants (MTHFR 1298A>C and 1781G>A) were protective. As mentioned above maternal polymorphisms related to B12 levels predicted risk of NTD in the multicenter study. This supports a causality for association of low maternal B12 with higher risk of NTDs in Indians.

Maternal hyperhomocysteinemia was associated with increased risk for congenital heart defects (38). A study on risk factors for orofacial clefts (OFC) reported exclusive vegetarianism (proxy for low B12 status) to be a significant risk factor. However folic acid supplementation in the first 3 months of pregnancy was not protective against OFC (when adjusted for family history of OFC) (39). These studies were rated to be of low quality.

#### Comment

Neural tube defects (NTD) are an important congenital anomaly of public health significance. Although, there are multiple causal factors to this condition, the protective role of maternal folate is well established. The GRADE evidence for association of low maternal vitamin B12 with greater risk of NTD outcome in offspring is high. This is supported by many observational studies and associations with genetic determinants of vitamin B12. The evidence suggests a role for maternal vitamin B12 in reducing the risk for NTD in offspring over and above the well-established role of maternal folate.

### Birth Size

Ten studies (23, 24, 41–44, 48–50, 70) reported birth size (neonatal anthropometry – length, head, abdomen, chest circumference and or birth weight), risk for small for gestational age (SGA), intrauterine growth retardation (IUGR) (**Supplementary Table 4**). Low birth weight (LBW) was defined as birth weight <2500gms. IUGR/SGA as birth weight below the 10th centile for gestational age at delivery. Four studies (40, 45–47) reported birth size outcomes in supplementation trials (**Supplementary Table 8**).

#### Observational Studies

One observational study measured dietary patterns during pregnancy (24-hour recall & food frequency questionnaire) at 18 and 28 weeks of pregnancy (41). Higher consumption of green leafy vegetables and fruits at 28-week pregnancy was associated with increase in birth weight of 19.4 g (p<0.001) and 7.4 g (p<0.01) respectively for every increased consumption of the food group by one time per week.

Five studies examined maternal blood levels of vitamin B12, folate and homocysteine during three trimesters of pregnancy and associations with birth size. A case control study (49) compared a subset of SGA with appropriate for gestational age babies from the Pune Maternal Nutrition Study (PMNS) birth cohort and found significantly higher maternal homocysteine levels at 28 weeks of gestation in the SGA group (7.2 µmol/L vs. 5.5 µmol/L). Maternal homocysteine was inversely associated with offspring birth weight. Muthayya et al. (43) followed up 112 pregnant women (serially in every trimester till delivery from 12th week of gestation) from urban slums of Bangalore (43). Eighteen percent neonates were LBW. Vitamin B12 concentration in mothers during pregnancy correlated with cord values. The associations were strongest for the second and third trimester. Neonates in birth weight categories (<2500gms and 2500-2999gms) had lower cord B12 values than the group with birth weight >3000gms (264 and 268 pg/ml vs. 320 pg/ml, p=0.02). A related paper (48) on the same study sample reported that offspring born to women in the lowest tertile of vitamin B12 levels had higher risk for IUGR (Effect was most pronounced in the second trimester – adjusted OR = 9.3, 95%CI 2.9, 29.7). Wadhwani et al. (24) examined 106 pregnant women prospectively (serially in every trimester till delivery) and reported that lower birth weight was associated with lower maternal folate and B12 (r=0.2, p<0.05) in the third trimester and higher maternal homocysteine (r=-0.2, p<0.05) in the first trimester (24). Mishra et al. (42) in a prospective follow up of 100 pregnancies in a tertiary care centre in North India (Delhi) observed vitamin B12 deficiency in the first trimester to be associated with low birth weight outcomes (OR = 8.1) (42).

Two studies (23, 70) reported effects of imbalance in maternal vitamin B12 to folate. A cross-sectional observation study from Pune on 49 pregnant women found no association between maternal vitamin B12 and folate (measured at 36 weeks of gestation) with any of the neonatal anthropometry measures. However, a higher maternal folate to B12 ratio was associated with higher maternal homocysteine, smaller birth weight (r=-0.5, p=0.009), length (r=-0.4, p=0.03), chest circumference (r=-0.5, p=0.009) and head circumference (r=-0.4, p=0.02). Dwarkanath et al. (50) prospectively examined a cohort of 1838 women from urban slums of Bangalore for dietary micronutrient intakes (in each trimester). They reported a 30% prevalence of SGA in their cohort. Low dietary intake of vitamin B12 and folate rich foods was associated with higher risk for SGA babies (RR=1.2). In a subgroup of women that received high folic acid supplements (>1000mcg/d) during the second trimester, the lowest tertile of B12 to folate intake ratio had highest risk for SGA babies (RR=2.73).

One study (71) examined the association of between maternal genetic polymorphism of folate metabolism with birth weight. Mothers with the MTHFR gene variant (C677T) had significantly elevated homocysteine levels and lower birth weight in their offspring. Maternal risk genotype at rs1801133 predicted higher homocysteine concentration and lower birth weight (B=-61, 95%CI: -111, -10). Using a Mendelian Randomization technique, a causal association between maternal homocysteine and low birth weight in offspring was demonstrated. In another study maternal global DNA methylation (epigenetic markers) showed significant correlation with total plasma homocysteine in the vegetarian mothers which in turn was associated with offspring’s chest circumference and triceps skin fold thickness at birth (r=-0.39, p=0.04).

#### Intervention Studies

A placebo controlled Randomized Controlled Trial (RCT) (40) in a tertiary hospital in Delhi studied 200 undernourished pregnant women who received MMN (29 micronutrients including 1mcg of B12) in their third trimester. Infants in the micronutrient supplemented group were heavier by 98gms. The incidence of low birth weight decreased from 43.1% to 16.2% (RR=0.3, 95%CI 0.13, 0.71) and early neonatal mortality decreased from 28% to 14.8% (RR=0.42, 95%CI 0.19, 0.94) in the supplemented group. Observations from the MMNP (45) report that in ~1300 live births, there was no overall effect of pre-conceptional food based intervention (micronutrient snack of green leafy vegetables and milk powder) on ultrasound measures of fetal size (46) or birth weight. In a subgroup of mothers who received intervention more than 90 days before pregnancy there was an increase in birth weight by 48 grams. There was a significant interaction between maternal pre-pregnancy BMI and supplementation. Offspring of mothers with higher pre-pregnancy BMI in the supplementation group showed a higher birth weight. The prevalence of LBW was similar between intervention and control groups (34 vs 39%). A randomized controlled trial from Bangalore (47) recruited 366 mothers randomized to receive either oral 50mcg B12 or placebo from first trimester of pregnancy (14 weeks). There was no difference in birth weight (primary outcome) between the intervention groups (2.85 ± 0.46 kg in the vitamin B12 group vs. 2.83 ± 0.45 kg in the placebo group).

### Comments

Observations in this review are supported by two meta-analyses of maternal vitamin B12 status and pregnancy outcomes. One meta-analysis of 18 studies world-wide reported a linear association between maternal vitamin B12 levels and preterm birth (RR of 0.89 per SD decrease in maternal B12) (14). Another meta-analysis (13) of 23 studies reported a higher risk for low birth weight (OR 1.7) in mothers with low vitamin B12 values (studies from India largely contributed to this outcome). Results from micronutrient supplementation trials in other low-income countries provide further evidence for a causal role of vitamin B12 in determining birth outcomes. A trial from Nepal (multi micronutrient containing 2.6mcg B12) showed higher birth weight by 64gms but did not reduce neonatal mortality (71). The Cochrane review (72) of 20 trials of MMN supplementation (19 done in low and middle-income countries – including Indian Subcontinent - Nepal, Bangladesh and Pakistan) concluded that there was a high quality evidence for reduction in LBW (RR=0.88 95%CI 0.85,0.91) and moderate quality evidence for reduction in risk of SGA (RR=0.92, 95%CI 0.88.0.97).

Lower maternal vitamin B12 status or high homocysteine or higher folate to B12 ratios during pregnancy was associated with increased risk for LBW, SGA and IUGR. The associations were strongest for concentrations in the second trimester. The study by Yajnik et al. (44) used a technique of Mendelian randomization to support a causal association of higher maternal homocysteine with lower birth weight using MTHFR C677T polymorphism. Though the study did not directly examine genetic determinants of B12; in that population, hyper homocysteinemia was attributable to deficiency of B12 rather than folate. One supplementation study (40) showed a large effect for reduction in LBW with MMN supplements (inclusive of vitamin B12) in severely undernourished women.

The GRADE evidence for associations of low maternal vitamin B12 or high homocysteine (as marker of B12 deficiency) with low birth weight is high. The GRADE evidence for risk of IUGR and SGA is moderate. The level of evidence for other neonatal size (length, head, chest, abdomen circumference) is low. The evidence supports an association between high prevalence of vitamin B12 deficiency in pregnancy and high incidence of low birth weight in India.

### Later Life Health Outcomes in the Offspring

#### Offspring B12 Status

Three studies reported child vitamin B12 levels in association with maternal vitamin B12 status (**Supplementary Table 5**). Finkelstein et al. (21) reported that impaired vitamin B12 status (low maternal vitamin B12 and high MMA) in pregnancy was associated with lower infant B12 levels at 6 weeks (adjusted for B12 intervention). The strongest associations were reported with values in the third trimester (p<0.01). Infants born to women with vitamin B12 deficiency had a twofold greater risk of vitamin B12 deficiency (P<0.01). Higher maternal folate concentrations also predicted lower risk of vitamin B12 deficiency in infants (P<0.05). Lubree et al. (51) reported that higher maternal B12 at 34 weeks, gestation was associated with a higher child vitamin B12 and lower homocysteine at 2 years of age (B=0.2 and -0.2 respectively, p=0.02 adjusted for current diet of child, breastfeeding, cord B12 and folate). Christian et al. (52) reported in a prospective cohort from Mysore, that child’s vitamin B12 concentrations at 9.5 years of age was associated with maternal pregnancy vitamin B12, independent of current dietary intake patterns of B12 (Increase in child vitamin B12 by 0.22% for every unit increase in maternal B12, 95%CI 0.14, 0.3).

#### Metabolic and Cardiovascular Functioning

One study (53) reported bone mineral density in offspring. Four studies (19, 54–56) reported maternal and birth characteristics in relation to offspring metabolic and cardiovascular outcomes and all of them were performed on ongoing longitudinal prospective birth cohorts (**Supplementary Table 6**).

##### Observational Studies

One study (53) on the PMNS birth cohort examined maternal circulating micronutrients (red cell folate, B12, vitamin C and ferritin) during pregnancy and bone mass in offspring at 6 years of age. Children of mothers who had higher frequency of intake of milk products, green leafy vegetables and non-veg foods and higher folate status at 28-week gestation had higher total (B=0.13, p<0.01) and spine bone mineral density (B=0.17, p<0.001) adjusted for parental size and fat mass. There was no association with maternal vitamin B12 status. Two studies examined maternal levels of vitamin B12, folate and homocysteine during pregnancy with metabolic outcomes in offspring in childhood in prospective birth cohorts. The Pune Maternal Nutrition Study (PMNS) reported outcomes at 6 years (19) and found lower maternal vitamin B12 at 18 weeks and higher maternal erythrocyte folate at 28 weeks to be associated with greater insulin resistance in the offspring (B=-0.16, p=0.03 and 0.38, p<0.001). Higher maternal erythrocyte folate was additionally associated with higher offspring adiposity (p<0.01). Children born to mothers with a combination of high folate and low B12 during pregnancy were most insulin resistant. The (54) Mysore Parthenon birth cohort reported that higher maternal homocysteine at 30 weeks gestation was associated with higher glucose concentrations in the child at 5 years (B=0.17, p=0.007), and higher folate in the third trimester of pregnancy was associated with greater insulin resistance in offspring at 9.5 years (B=0.1, p=0.03, 95%CI 0.01, 0.2). Maternal vitamin B12 concentrations were unrelated to offspring outcomes.

Another study (55) from the Mysore cohort examined cortisol and cardiovascular responses to a stress test in adolescents (at 13.5yrs) and found that vitamin B12 deficiency in mothers and a higher homocysteine was associated with a greater cortisol response (B=0.36 95%CI 0.16, 0.57) and greater heart rate response (B=0.13, p=0.01) respectively. A study (56) in offspring aged 3 to 8 years, examined cardiac autonomic nerve activity as measured by heart rate variability (HRV) in relation to maternal vitamin B12 status. Children born to mothers with a low vitamin B12 status (<114pmol/L) showed a 53% reduction in the HRV measured in the low-frequency bands compared to children born to mothers with a higher B12 status. Higher low-frequency HRV in children was also associated with higher cord B12 levels (p=0.03).

#### Cognitive Functioning

##### Observational Studies

Three observational studies reported neurocognitive outcomes in relation to maternal B12, folate and homocysteine during pregnancy (**Supplementary Table 7**). Three studies (57–59) examined maternal micronutrient status as part of ongoing longitudinal cohort studies at Pune and Mysore. One study (57) from Pune reported that lower maternal B12 and folate concentrations at 28 and 34 weeks, gestation were associated with lower scores on mental and social development (independent of offspring B12 and folate status). In the PMNS cohort offspring of mothers in the lowest decile of maternal vitamin B12 levels at 28 weeks pregnancy (<77pM) had lower performance on tests for attention and executive functions at 9 years (58) compared to offspring of mothers in the highest decile. In the Mysore cohort, higher maternal folate at 30 weeks pregnancy was associated with better cognitive scores in the child at 9.5 years (B = 0.1 to 0.2), with no associations with maternal B12 or homocysteine (59).

##### Intervention Studies

Two intervention studies (60, 61) reported an effect of B12 supplementation on offspring cognitive status at 9 months and 30 months of age in the context of a randomized controlled trial (**Supplementary Table 8**). In this trial, mothers were randomized to receive either 50mcg oral vitamin B12 supplementation or placebo from the 1^st^ trimester of pregnancy till 6 weeks postpartum. Early childhood cognitive development was assessed on Bayley Scales of Infant Development (BSID). At 9 months (61), there was no difference between the two supplemented groups on BSID domains but elevated homocysteine in mothers during the second and third trimester of pregnancy was negatively associated with expressive language (B=-0.3, 95%CI -4.13, -2.14 adjusted for birth weight, home environment, economic status) and motor functions. At 30 months of age (60), children of mothers in the B12 supplemented group had significantly higher scores on expressive language (36.01 vs 34.78, p=0.03). Higher maternal homocysteine continued to predict lower expressive language functions at 30 months though the strength of association had reduced (adjusted B= -0.18, p=0.03). The effect of vitamin B12 supplementation on language at 30 months corresponds to the developmental milestones for language functions.

##### Comment

These observations are supported by findings from neighboring Nepal. A study by Stewart et al. (73) reported that low maternal vitamin B12 is associated with higher insulin resistance at 6-8 years of age regardless of antenatal micronutrient supplementation. Meta-analysis from western studies highlight the role of maternal obesity in offspring metabolic and cognitive outcomes (74, 75). A Mendelian randomization study in the ALSPAC UK birth cohort examined maternal vitamin B12 dietary intakes and cord vitamin B12 measurements and found weak causal associations with offspring intelligence (76). However maternal vitamin B12 concentrations were not evaluated and sample size with cord B12 measurements was small.

In India, maternal undernutrition is widely prevalent. A fetus born to an undernourished mother is programmed for an increased risk of adverse health outcomes in later life (77). This may explain the rising epidemic of Non-Communicable Diseases (NCD’s) such as diabetes and cardiovascular disorders. The maternal nutritional observations in India are limited to cohorts from western (Pune, Mumbai) and southern parts (Mysore, Bengaluru) of India. Observations from other geographical regions will be of interest to inform whether the observed associations of maternal vitamin B12 and offspring health outcomes are generalizable to the whole Indian population. Birth cohorts from Vellore and Delhi have reported associations between fetal growth and risk for NCD in later life (78, 79). However, they did not measure maternal nutritional status. Out of the long-term outcomes, evidence from intervention trials is available only for neurocognitive functions. There is a need to establish causality of associations using randomized controlled trials or Mendelian randomization techniques for other long-term outcomes as well. The GRADE evidence for later life health outcomes (adiposity, insulin resistance) is of a moderate level. The evidence is higher for cognitive functions which is supported for causality by a randomized controlled trial.

## Discussion

The reported prevalence rates of vitamin B12 deficiency (plasma concentrations <150pM) ranges from 40-70%. These rates are reported from south, west and north India. Vitamin B12 measurements were markedly lower in the third trimester of pregnancy as compared to early pregnancy. Rates of folate deficiency (20-40%) were lower than vitamin B12 deficiency and comparable to worldwide estimates. Low maternal vitamin B12 was associated with a higher risk of NTD in the offspring, and increased risk for poor fetal growth reported as LBW, SGA or IUGR. In addition, low maternal vitamin B12 was also associated with lower offspring B12 concentrations in cord blood as well as during childhood, and with poor neurocognitive development and increased risk for diabetes (insulin resistance in childhood). An imbalance in B12-folate status (low B12-high folate) was associated with a higher risk for gestational diabetes and subsequent permanent diabetes, greater insulin resistance, and adiposity in the offspring during childhood. Maternal hyper-homocysteinemia (a marker of vitamin B12 deficiency) was associated with a higher risk for recurrent pregnancy losses, preeclampsia and low birth weight. Supplementation studies support a beneficial effect of antenatal B12 supplementation on neurocognitive development in the offspring. Micronutrient supplementation in undernourished pregnant mothers (inclusive of vitamin B12) resulted in increased birth weight.

**Figure 2:**
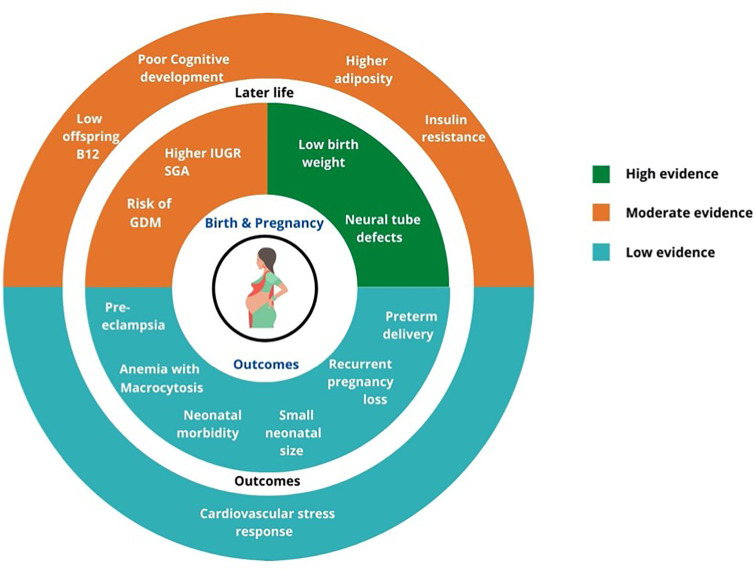
Summary of GRADE evidence for studies on associations of low maternal vitamin B12 and pregnancy and offspring health outcomes.

From the GRADE rating (**Figure 2**), the quality of evidence for role of lower maternal vitamin B12 is high for risk of NTD and low birth weight in offspring, moderate for risk of gestational diabetes, IUGR and SGA, and later life health outcomes (lower childhood vitamin B12 status, higher adiposity and insulin resistance and poorer neurocognitive functions), and low for risk of preterm delivery, preeclampsia, and recurrent pregnancy losses.

In the category of high-quality evidence, neural tube defects (NTD) are important congenital anomalies of public health significance because of severe disability and cost of management. India has a high burden of NTDs. Cherian et al. (80) reported a prevalence of NTD of 6.57 to 8.21 per 1000 live births in rural Uttar Pradesh, before the days of ultrasound screening. Godbole et al. (81) in a review reported an incidence varying from 0.5 to 11/1000 live births in different studies in India. Thankfully, since the widespread use of antenatal ultrasound, such pregnancies are medically terminated and therefore have reduced the incidence in live babies.

India is the world’s capital for the burden of low birth weight babies (82). Studies reporting the association between low maternal vitamin B12 and low birth weight had a high rating. Low birth weight makes a substantial contribution to perinatal morbidity and mortality. It will be important to study the effect of maternal vitamin B12 supplementation on this important aspect of public health. In addition recent research has highlighted the role of low birth weight in increasing the risk of non-communicable diseases (DOHaD theory) (83). Observations from prospective birth cohorts in India have reported associations of low birth weight with increased risk for cardiovascular and metabolic disorders in adulthood (diabetes, hypertension, coronary artery disease) (78, 84). This is a novel explanation for the escalating epidemic of NCD’s in India and provides a rationale for the observation that Indians get NCD’s at a younger age and a lower BMI compared to Europeans (3, 85). Rapid postnatal growth and increase in BMI (even though in the normal range) exaggerates the risk of diabetes and related disorders. Optimizing maternal nutrition (prevention and treatment of anaemia, correcting micronutrient deficiencies such as vitamin B12) in women in the reproductive age group may be a useful strategy to reduce the incidence of low birth weight and the risk of NCD in the next generation.

### Mechanism for Effects of Maternal B12 and Folate on Fetal Growth

Vitamin B12 and folate are important for the methionine cycle (8, 86) where homocysteine is converted to methionine by methionine synthase. Methylcobalamin is the co-enzyme for this reaction. Simultaneously methyl-tetra-hydrofolate is converted to tetra-hydrofolate and the methyl group converts homocysteine to methionine which in turn generates SAM (S-adenosyl methionine). SAM is a universal methyl donor and supports many metabolic processes (DNA synthesis, stability, methylation which is an epigenetic mechanism). Vitamin B12 deficiency leads to a ‘folate trap’ where folate is trapped in the unusable methyl form and the methyl groups are not available for metabolic cycles (87). In clinical practice, folate is available as folic acid which is more stable than natural folates and has higher bioavailability. Folic acid needs to be reduced by liver metabolism. Un-metabolized folic acid has a higher affinity for folate receptors and could competitively inhibit natural folates from binding with the folate receptors (88). This may be particularly detrimental in the presence of vitamin B12 deficiency and could produce ill effects. In addition, hyperhomocystenemia could have an independent adverse effect on vascular endothelial function mediated through oxidative stress. This can impact the vascular system, and affect the placental function and fetal growth (89).

### Evolution of Policy on Micronutrient Supplementation in India

Lucy Wills from the Haffkine Institute Mumbai in 1931 first described the successful treatment of ‘tropical macrocytic anaemia’ in pregnancy with liver or yeast extract (marmite) (90). Subsequently, formal studies have been conducted since the 1970s. Yusufji et al. (91) showed that a substantial 52% of 1000 pregnant women in Vellore, South India had low B12 concentrations and 66% had megaloblastosis. WHO collaborative studies on nutritional anaemia (92) in India examined the effects of different doses of oral iron (30-240mg), with additional micronutrients in pregnant women from low socio-economic status groups at Delhi and Vellore. The best results were seen in groups receiving 120 and 240 mg of iron (+3.32 and +3.8g/100ml respectively) along with B12 and folate. The group receiving B12 and folate in addition to iron (120mg) showed a greater increase in haemoglobin than the group receiving iron (120mg) alone. The authors ascribed a greater increase in haemoglobin in the Iron+B12+folic acid group to the effect of folic acid rather than B12. This may be debated. There was no effect on birth weight or haemoglobin concentrations in the mother and infant 3 months after delivery. The authors recommend that a pregnant woman should have adequate levels of iron, folic acid and B12.

The Iron-folic acid (IFA) supplementation program in women in the reproductive age group and preschool children (1-5years) was initiated in India in 1970 to reduce the incidence of anaemia as part of the National Nutritional Anaemia Prophylaxis Programme. Pregnant and lactating mothers were prescribed 60mg elemental iron (later increased to 100 mg) and 500mcg folic acid for 100 days. In 2013 the program was modified as the National Iron Plus Initiative (NIPI) and in addition to pregnant and lactating mothers also covered children and adolescents (6 months to 19 years) and women of reproductive age. Adolescents (12-19yrs) and women in the reproductive age groups received an IFA tablet once a week (100mg iron + 500mcg folic acid) (Weekly Iron Folic acid Supplementation program - WIFS).

### Folic Acid Supplementation in India for Prevention of NTD

Another benefit of folic acid supplementation is to reduce the incidence of NTDs. This was demonstrated in the MRC Vitamin Study (93). In this multicentre study from 33 centres, ~1800 women with a previous history of NTD were randomized to four groups to receive 4mg folic acid, multivitamins, both or neither. In the 1195 pregnancies that occurred, there was a 72% reduction in risk of recurrent NTD in the mothers who received folic acid 4mg/day. The Hungarian trial (94) demonstrated a significant reduction in first occurrence NTD with peri-conceptional multivitamin supplementation (inclusive of folic acid 0.8 mg and 4mcg B12) started at least 1 month before conception. The Indian Council of Medical Research (ICMR) trial (95) to prevent recurrence of NTD used 4mg folic acid with MMN (without B12) in ~400 mothers with a history of NTD. The controls received iron and calcium. The trial was stopped on ethical grounds after the publication of the MRC trial. The intervention reduced the risk of recurrent NTD by 60%. The currently recommended dose of folic acid to prevent NTD is 400mcg/day starting before conception (WHO), and 4mg/day to prevent recurrent NTD (96).

The current IFA policy in India provides 500 mcg of folic acid once a week. There is a need to coordinate anaemia prophylaxis and NTD prophylaxis in national programs. On the other hand, it is a common clinical practice for obstetricians to prescribe 5mg folic acid tablets for intended prevention of NTDs, anaemia and sometimes preeclampsia. This has led to many combinations in the market where doses of 1mg or more are included. The most commonly used tablet in India contains 5mg (The safe upper limit (SUL) for folic acid in European countries is 1mg/day). It is noteworthy that the majority of pregnancies in India are unplanned and the first visit to a doctor is long after the neural tube has closed (or not closed by 28 days gestation) which fact is forgotten by prescribers of high dose folic acid. Discussion with some of the obstetricians also made us realise that there is only a limited appreciation that a higher folic acid dose (4mg) is only for the prevention of recurrent NTDs. This practice in a largely B12 deficient population inadvertently causes an imbalance of B12 and folate status. This is a potential action point for makers of policy and clinical practice guidelines.

### Gaps in Public Health Policy on Micronutrient Supplementation in India

Serial observations from the NFHS surveys (1993–2015) have shown a persistently high prevalence of anaemia in pregnancy (50-60%) (97). The prevalence of LBW has shown a reduction from (23% to 18%) (98) which may be ascribed to an increase in maternal height and weight in younger generations many social, economic and health system improvements. Despite these improvements, a high prevalence of anaemia in pregnancy remains a significant public health problem.

Limitations of current supplementation programs are 1) Supplementation in pregnancy is provided at the first antenatal visit, usually after the first trimester (14-16 weeks gestation). Important fetal developmental milestones such as placentogenesis, closure of the neural tube (28 days) and organogenesis are over by this time. Current standards of practice miss the important early developmental window 2) Current policy is a weekly supplementation of IFA to adolescents and women in the reproductive age group. Adequacy of this approach to satisfactorily improve stores of iron and folate is doubtful. 3) The focus of these public health measures is the control of anaemia. Lack of major effect on anaemia prevalence mandates a re-evaluation of possible contributory factors, including the addition of vitamin B12 and a series of logistic and health system-related factors. 4) Prevention of NTDs is still not an important component of the program

A recent review of the NIPI program (99) discussed recommendations to strengthen the program and suggested that there is a need to 1) Revise the dose, frequency and duration of the iron supplements. WHO guidelines 2016 (100) recommend a daily dose of elemental iron of 30-60 mg for 3 consecutive months in a year for women in the reproductive age group. 2) Correct other micronutrient deficiencies such as vitamin B12 that contribute to anaemia. 3) Use fortified foods (such as salt and ultra-rice) as an adjunct or alternate supplementation strategy. Presently the Government of India is implementing the National Nutrition Mission which accords high priority to reducing rates of anaemia in children and women of reproductive age and low birth weight. This mission now incorporates the Intensified – NIPI (I-NIPI) program initiated in 2018 (101). The dose of elemental iron to adolescents and women in the reproductive age group has been reduced to 60mg to improve tolerability (gastric side effects with 100mg), however, the frequency continues to be weekly. Pregnant women will receive supplementation for 180 days and another 180 days postpartum. Four hundred mcg folic acid tablets daily are advised during the pre-conceptional period and first trimester to reduce the incidence of neural tube defects. The Government of India has mandated the use of fortified salt (iodine and iron), oil (vitamin A and D), wheat and rice (iron, folic acid, vitamin B12) in foods served under Integrated Child Development Services (ICDS) and Mid-day Meal (MDM) schemes to address micronutrient deficiencies in children. The I-NIPI program aims to reduce the prevalence of anaemia in pregnancy from ~50% to 32% in the next 3 years.

### A Life Course Approach to Improving Nutrition

Supplementing vitamin B12 at near RDA dose to children, adolescents, and women in the reproductive age group and lactating mothers may be a prudent strategy to improve maternal and child health. The RDA dose for B12 in India for women is 2.5mcg/day (102). The timing of intervention is an important factor for the supplementation to be effective. Supplementation beginning in childhood, adolescence and continuing in the pre-conceptional period, pregnancy and lactation would ensure adequate micronutrient stores not only in the pre-and peri-conceptional period but also for the growing infant. The period around conception is now recognized to be crucial for mediating parental influences on the health of the next generation (peri-conceptional epigenetic reprogramming). Maternal nutrition and lifestyle interventions in this important window period are now a focus of public health policy worldwide (103). This ‘life course approach’ to improve the health of individuals would help break the intergenerational cycle of transmission of risk for NCDs to the next generation.

Our group has recently concluded a pre-conceptional vitamin B12 intervention trial with 2mcg oral B12/day or B12 2mcg/day+MMN or placebo in participants of the Pune Maternal Nutrition Study (PRIYA trial) (104). The study has been recently un-blinded and early results from the trial (unpublished) are promising and show a beneficial effect of supplementation on improving maternal B12 status, cord vitamin B12 status (primary outcome) and a reduction in homocysteine levels. The results also show a benefit of intervention on neurocognitive development in the offspring at 2 years of age. In another study (105) on rural adolescent women with severe vitamin B12 deficiency (B12<100pM), we demonstrated a significant improvement in vitamin B12 status and nerve function with 11 months supplementation of 2mcg B12/day.

### Recommendations

Based on the quality of evidence reviewed, we recommend the addition of vitamin B12 to various nutritional programs in India for children, adolescents, women in the reproductive age group, pregnant and lactating women. This public health measure would help in reduction in risk of NTD and improving birth weight in the offspring. Clinical guidelines should discourage the use of a high dose of folic acid in women of reproductive age and mothers without a history of previous NTD. If a high dose of folic acid is indicated it should be accompanied with vitamin B12.

### Strength and Limitations

We performed an extensive literature search and cross-referencing including the IndMED database for studies published in Indian journals and not indexed elsewhere. Data were extracted and reported as per the PRISMA guidelines. Since, there were methodological variations (study design, timing of assessment of exposures) even in studies reporting similar outcomes, we were unable to perform a meta-analysis. All studies were reviewed by two independent raters and rated for quality of evidence on the GRADE. We examined and reported causal associations where evidence was available. Using the GRADE system enabled us to objectively rate the quality of evidence to inform policy.

## Conclusion

This review confirms a wide prevalence of vitamin B12 deficiency in Indian women during pregnancy. Vitamin B12 and folate act as methyl donors in one-carbon metabolism which affects cell growth and differentiation by influencing DNA synthesis and epigenetic regulation. Therefore, in addition to the well-recognized hematological benefits they are important regulators of fetal growth and development. Studies from India demonstrated causative associations between maternal vitamin B12 deficiency and incidence of NTD and between vitamin B12 associated hyperhomocysteinemia and low birth weight. Observational studies also demonstrate an association of low maternal vitamin B12, higher homocysteine or imbalance between B12 and folate status with risk of pregnancy complications (recurrent pregnancy losses, gestational diabetes, pre-eclampsia), SGA, IUGR and adverse longer-term health outcomes in the offspring (cognitive functions, adiposity, insulin resistance). This may be addressed by supplementation of near RDA doses of vitamin B12 in addition to the well-established IFA supplementation, in a life-course model from early childhood into the reproductive age. Clinicians need to be educated about the possible detrimental effects of high dose folic acid in vitamin B12 deficient populations.

## Data Availability Statement

The original contributions presented in the study are included in the article/**Supplementary Material**. Further inquiries can be directed to the corresponding author.

## Author Contributions

CY conceptualized the need for review and search strategy. RB and AD performed the search, extraction of results, and reviewed the studies for quality of evidence. RB wrote the first draft. SO contributed to manuscript writing. MD and CY supervised the grading for quality of evidence, formulating recommendations, and edited the manuscript. All authors contributed to the article and approved the submitted version.

## Funding 

This work is supported by DBT/Wellcome Trust India Alliance Fellowship [IA/CPHI/161502665] awarded to RB, DBT-CEIB grant BT/PR12629/MED/97/364/2016 awarded to CY, and Biotechnology and Biological Sciences Research Council (BBSRC) grant BB/SO14020/1 awarded to CY.

## Conflict of Interest

The authors declare that the research was conducted in the absence of any commercial or financial relationships that could be construed as a potential conflict of interest.
