# Supplementary material for: Maternal Vitamin B12 Status During Pregnancy and Its Association With Outcomes of Pregnancy and Health of the Offspring: A Systematic Review and Implications for Policy in India
Source: Front Endocrinol (Lausanne). 2021 Apr 12;12:619176. doi: 10.3389/fendo.2021.619176 (PMC8074968; doi:10.3389/fendo.2021.619176)
Supplement: Supplementary file 1 [file Table_1.pdf]

Supplementary Table – 1 Indian studies on B12 during pregnancy and anemia

**Macrocytosis with anaemia (2 studies)**					
**Sl. No.**	**Paper/ Author/ Year**	**Exposure**	**Test/Procedure**	**Outcomes**	**Results**
1	Anemia of pregnancy in northern India. Nature and therapeutic follow-up<br><br>Khanna et al., 1977 (25)	Pregnancy	114 anaemic pregnant women	Anemia	Megaloblastosis occurred in more than half<br><br>Vitamin B12-deficiency was fairly common<br>All patients with normoblastic marrow achieved full remission with iron therapy alone<br><br>The deficiency of haematinic factors was largely due to poor nutrition
2	Clinical evaluation of severe anemia in pregnancy with special reference to macrocytic anemia<br><br>Tripathi et al., 2012 (26)	Severe anemia in pregnancy	All antenatal patients presenting with severe anemia (hemoglobin <7gm%) over a period of 2years from January 2008 to December 2009	Haematological profile of women with anaemia	High prevalence of macrocytic anemia (40%)<br><br>The maternal and fetal morbidity and mortality is higher in macrocytic anemia as compared to iron-deficiency anemia

Supplementary Table – 2 Indian studies on B12 during pregnancy and maternal pregnancy complications

Maternal pregnancy complications (7) studies					
**Sl. No.**	**Paper/ Author/ Year**	**Exposure**	**Study design, Subjects, Test/Procedure**	**Outcomes**	**Results**
1	Association of omega-3 fatty acids and homocysteine concentrations in pre-eclampsia<br><br>Kulkarni et al., 2011 (29)	Maternal folate, vitamin B_{12}, omega-3 fatty acids and homocysteine levels measured just before delivery	Case control design 49 Pre-eclamptic and 57 normotensive women.	Pre-eclampsia	Homocysteine concentrations were higher in pre-eclamptic than in normotensive women (14.28 vs 11.03umol/L, p<0.01) despite similar levels of folic acid and vitamin B_{12}
2	Study of Serum Homocysteine, Folic Acid and Vitamin B_{12} in Patients with Preeclampsia<br><br>Mujawar et al., 2011 (30)	Occurrence of association between homocysteine, folic acid and vitamin B12	Fifty preeclamptic patients from gynecology ward were studied for estimation of serum homocysteine, folic acid and vitamin B12<br>Control group consist of 50 healthy normotensive pregnant subjects of identical age without any disease	Preeclampsia	Preeclampsia group had higher homocysteine(16.4vs 8.19umol/L, p<0.001) lower B12 (321 vs 391 pg/ml, p<0.001) and folate levels compared to the control group.

3	Reduced Folate, Increased Vitamin B 12 and Homocysteine Concentrations in Women Delivering Preterm<br><br>Dhobale et al., 2012 (31)	Maternal levels of folate, vitamin B 12 and homocysteine in mothers just before delivery	Case control design. 76 women delivering at term (control group), 67 women delivering preterm (PT group) and 49 women with preeclampsia delivering preterm (PT-PE group) with singleton pregnancy	Preterm delivery, Preterm delivery due to preeclampsia or Term delivery in normotensive women	Increased vitamin B 12 and homocysteine levels were seen in the PT-PE and PT groups as compared to the controls.<br><br>Reduced folate levels were observed in the PT group<br><br>A negative association of maternal plasma Homocysteine with birth weight was seen in the PT group (r=-0.3)
4	MTHFR C677T polymorphism, folate, vitamin B12 and homocysteine in recurrent pregnancy losses: a case control study among North Indian women<br><br>Puri et al., 2013 (27)	Maternal Methylenetetrahydrofolate reductase genotype (MTHFR C677T) in relation to homocysteine, B12, folate levels.	107 women with three or more consecutive unexplained recurrent pregnancy losses and 343 women with two or more successful and uncom- plicated pregnancies	Recurrent pregnancy losses	MTHFR genotypic distribution among cases and controls showed no significant difference<br><br>MTHFR C677T polymorphism was found to be significantly associated with increased homocysteine in the case group. Hyperhomocysteinemia (OR = 7.02, 95%CI 3.8 – 12.8) and vitamin B_{12} deficiency (OR=16.39, 95%CI 7.7 – 34.8) were significant risk factors for recurrent pregnancy loss

5	Recurrent pregnancy losses vis-à-vis anemia and vitamin (Folate/ B12) imbalance<br><br>Kaur et al., 2018 (28)	Anemia, folate and B12 imbalance	Case control study of 105 women with history of Idiopathic recurrent pregnancy loss and 135 Multiparous non pregnant women with 2 or more consecutive normal obstetric outcomes from a tertiary hospital setting in North India	Recurrent pregnancy losses (RPL)	Controls had folate deficiency anemia while non anaemic women with RPL had higher vitamin B12 deficiency (58%)<br><br>Non anemic RPL women had lowest B12/folate ratio (18.46 vs 124 in anemic controls, $p<0.001$)<br><br>A higher allelic frequency for C677T allele was observed (0.16 versus 0.11) in the case group
6	Higher maternal plasma folate, vitamin B12 and homocysteine levels in women with preeclampsia<br><br>Pisal et al., 2019 (32)	Maternal plasma folate, vitamin B12 and homocysteine levels at delivery	Case control study on 450 normotensive control and 350 women with preeclampsia from tertiary hospital in Pune<br><br>Preeclampsia defined as systolic and diastolic blood pressures greater than 140 and 90 mmHg, with	Preeclampsia and preterm delivery	Maternal vitamin B12, folate, deficiency was higher in the preeclampsia group as compared to the control group (41% vs 60%, $p<0.05$). Women with preeclampsia and preterm delivery had higher B12 and lower homocysteine.<br><br>Higher maternal homocysteine was associated with higher maternal systolic ($r=0.15$, $p< 0.001$)) and diastolic ($r=0.2$, $p<0.001$) blood pressure.

			presence of proteinuria		
7	Low plasma vitamin B12 in pregnancy is associated with gestational 'diabesity' and later diabetes<br><br>Krishnaveni et al., 2009 (18)	Maternal homocysteine, Vitamin B12, and folate concentration in third trimester of pregnancy	654 women from Mysore Parthenon cohort study assessed at 30 weeks' gestation on 100gm oral glucose tolerance test. Retested by OGTT after 5 year of delivery	Gestational Diabetes and Diabetes mellitus 5 years after delivery	B12 deficient women had higher incidence of GDM (OR = 2.1 95%CI 1.1, 3.6, adjusted for maternal body size, socioeconomic factors, familial risk of diabetes). Among B12 deficient women incidence of GDM increased with increasing folate concentrations (5.4% to 10.9%). B12 deficiency during pregnancy was associated with increased diabetes prevalence 5 years after delivery.

Supplementary Table – 3 Indian studies on B12 during pregnancy and congenital anomalies

**Offspring outcomes at birth: congenital anomalies* (6 studies)**					
**Sl. No.**	**Paper/ Author/ Year**	**Exposure**	**Test/Procedure**	**Outcomes**	**Results**
1	Evaluation of the levels of folate, vitamin B12, homocysteine and fluoride in the parents and the affected neonates with neural tube defect and their matched controls<br><br>Ratan et al., 2008 (34)	Folate, vitamin B12, and homocysteine levels in parents and neonates	Case control study in 35 neonates with NTD and their parents, 31 neonates with congenital anomalies and 24 neonates with no anomalies from north India.	Neural Tube Defects in Neonates	Neonates with NTD had higher homocysteine (17.9µmol/L vs 11.9µmol/L, $p=0.02$)<br><br>Lower RBC folate in mothers had higher odds for NTD (OR = 23.3, 95%CI 2.6-207.7)<br><br>High homocysteine in fathers was identified as an independent risk factor for NTD (adjusted OR = 26.5, 95%CI 2.6-262.4)
2	Folate supplementation, MTHFR gene polymorphism and neural tube defects: a community based case control study in North India<br><br>Deb et al., 2011 (35)	Potential role of MTHFR gene polymorphism, folate supplementation and dietary pattern	Mothers of NTD neonates (n=222) and controls (n=111) in heterogeneous populations of North India, with the	NTD	Not receiving folate supplements and vegetarian diet in mothers was associated with increased risk for NTD (OR = 1.92,

			special focus on their ethnic labels		95%CI 1.09-3.4 & OR = 1.77, 95%CI .02-3.05 respectively)
3	Maternal one-carbon metabolism, MTHFR and TCN2 genotypes and neural tube defects in India<br><br>Godbole et al., 2011 (37)	Mothers of offspring with NTD and healthy controls	Multicentric study of 318 women with NTD-affected offspring (cases) and 702 women with apparently healthy offspring (controls). Antenatal confirmation of NTD by fetal ultrasound	Comparison of plasma folate, vitamin B (12), homocysteine and holo-transcobalamin levels, and polymorphisms in methylenetetrahydrofolate reductase and transcobalamin levels	Cases (NTD) had significantly lower maternal holo-transcobalamin and higher maternal homocysteine levels. Prevalence of holo-transcobalamin was reported to be 65.3% vs 55.2% in NTD versus controls (p=0.003).<br><br>1298A>C and 1781G>A polymorphisms in MTHFR were protective<br><br>Maternal 776C>G polymorphism in TCN2 was strongly predictive of NTD in the offspring
4	Maternal hyperhomocysteinemia and congenital heart defects: A	Maternal serum homocysteine levels measured 9 to 15	Case control study of 30 women with neonates with congenital heart	Congenital heart disease in their offspring	46.6% cases had a tHcy level more than 15µmol/l compared

	prospective case control study in Indian population.<br><br>Malik et al., 2017 (38)	months after delivery	disease and 20 control women with healthy neonates		to 15% control mothers.
5	Risk factors for orofacial clefts in India: A case-control study<br><br>Neogi et al., 2017 (39)	Folic acid supplementation during the peri-conceptional period	785 participants were included in the study (157 cases and 628 controls)	Orofacial clefts in offspring	Exclusive vegetarianism in mothers associated with OFC (adjusted OR, 4.47; 95% CI, 1.83–10.98). Folic acid supplementation with folic acid during first 3 months of pregnancy was not protective.
6	Homocysteine and vitamin B12: Other causes of neural tube defects in Eastern Uttar Pradesh and Western Bihar population<br><br>Gupta R et al., 2018 (36)	Maternal and neonate's folic acid, homocysteine, and vitamin B12 levels	Retrospective Case control study<br><br>96 mothers who either had a first NTD child or had a history of NTD child in the family and 126 neonates with spina bifida from eastern India.<br><br>84 control mothers whose previous and current pregnancies were normal, and 87	Risk of development of NTDs	Folic acid level in the mothers and neonates was within the normal limit<br><br>NTD mothers had significantly raised homocysteine (14.57 vs 4.4 umol/L p<0.001) and low B12 levels (191.8 vs 478 pg/ml, p=0.002).

			control neonates who had no defects and were within the same age range as the cases<br>Samples in neonates collected within 1 to 4 weeks of life		

Supplementary Table – 4 Indian studies on B12 during pregnancy and birth size

Offspring outcomes at birth: birth size* (10 studies)					
**Sl. No.**	**Paper/ Author/ Year**	**Exposure**	**Test/Procedure**	**Outcomes**	**Results**
1	Intake of micronutrient-rich foods in rural Indian mothers is associated with the size of their babies at birth: Pune Maternal Nutrition Study<br><br>Rao et al., 2001 (41)	Maternal dietary intakes of macro and micro nutrients	Prospective study of 797 rural Indian women from Western India (Pune).<br>Maternal dietary intakes assessed by<br>24-h recall and food frequency questionnaire at<br>18 & 28 weeks of gestation	Birth size (Birth weight, length, head circumference, skin fold thickness)	Birth size was strongly associated with the consumption of milk at wk 18 and of green leafy vegetables (B=19.4, $p<0.001$) and fruits at wk 28 of gestation<br>Erythrocyte folate at 28 wk gestation was positively associated with birth weight. (Associations adjusted for gender, gestational age, social class, pre pregnancy weight, energy intake and physical activity)
2	Maternal total homocysteine	Maternal circulating concentrations of total	Mothers of full term small for gestation age (SGA)	Offspring birth weight	Mothers of SGA babies had higher plasma tHcy

	concentration and neonatal size in India<br><br>Yajnik CS et al., 2005 (49)	homocysteine (tHcy), vitamin B12 and folate at 28 weeks of pregnancy	babies (n=30) and appropriate for gestational age babies (n=50) selected from the Pune Maternal Nutrition Study Cohort.		concentration at 28wks gestation, than AGA babies(7.2 vs 5.5 umol/L, p<0.01).<br>Higher plasma tHcy concentrations were significantly associated with lower maternal B12 levels and lower birth weight (r=-0.28, p<0.05 adjusted for gender, maternal height and gestational weight at delivery)
3	The relationship of neonatal serum vitamin B12 status with birth weight<br><br>Muthayya et al., 2006 (43)	Maternal B12 during each trimesters of pregnancy and Cord B12 levels	410 women completed study with known pregnancy outcome. 112 women with live births and B12 measurements at each trimester and Cord B12 included in this analysis.	Birth weight. Offspring categorised into 3 groups based on birth wt (<2500 gms, upto 2999 gms and > 3000 gms)	B12 concentration in mothers during pregnancy correlated positively with cord values. Cord B12 was associated with birth weight upto 40 wks gestation (r=-0.3, p=0.01) . The associations were strongest for second and third trimester. Neonates in birth weight categories (<2500gms and 2500-2999gms) had lower cord B12 values than the group with birth weight >3000gms (Mean difference 52pg/ml)
4	Low maternal vitamin B12 status is associated with	Maternal sociodemographic, anthropometric, dietary	Prospective observational study from Bangalore. 478 mothers recruited at first	Birth weight in 377 live births<br>Prevalence of IUGR	28.6% of new born were IUGR

	intrauterine growth retardation in urban South Indians<br><br>Muthayya et al., 2006 (48)	and micronutrient status in apparently healthy pregnant women	trimester of pregnancy and followed up at every trimester. Dietary intake assessed on food frequency questionnaire. Erythrocyte folate levels.	- birth weight below the 10th centile of the gestational age according to the standards of the World Health Organization	Women in Lowest tertile of Vitamin B12 levels had highest odds for higher prevalence of IUGR in all trimesters. Effect was most pronounced in second trimester (OR = 9.28, 95%CI 2.9-29.68) adjusted for age, education, first trimester weight, parity). There was no association with erythrocyte folate.
5	High folate and low vitamin B-12 intakes during pregnancy are associated with small-for-gestational age infants in South Indian women: a prospective observational cohort study<br><br>Dwarkanath et al., 2013 (50)	Dietary intake of B12 and total folate and folic acid supplementation during pregnancy. Ratio of vitamin B-12 intake to total folate intake (folic acid supplement + dietary folate)	Prospective observational cohort study of 1838 pregnant women aged 17–40 y and at < 13 wk of gestation<br><br>Dietary intake of B12 and total folate and folic acid supplementation during pregnancy.<br>at baseline and in the 2 & 3rd trimester<br>Anthropometric measurements of the infants	Outcomes in small for- gestational-age (SGA) infants	Low B12 and folate dietary intakes were associated with higher risk for Small for Gestational Age babies (SGA) (RR=1.2). In a subgroup of women that received high folate supplements (>1000mcg/d) during second trimester the lowest tertile of B12 to folate intake ratio had highest risk for SGA babies (RR=2.73, 95%CI: 1.17, 6.37).
6	Maternal homocysteine in pregnancy and offspring birth weight:	Maternal total homocysteine	Tested for evidence of causality within a Mendelian randomization framework, using a	Offspring birth weight in the Pune Maternal Nutrition Study and Parthenon Cohort Study	Offspring birth weight was inversely related<br>to maternal homocysteine concentration adjusted for gestational age and offspring

	epidemiological associations and Mendelian randomization analysis<br><br>Yajnik CS et al., 2014 (44)		methylenetetra-hydrofolatereductase (MTHFR) gene variant rs1801133 (earlier known as 677C→T) by instrumental variable and triangulation analysis, separately and using meta-analysis		gender (B=-40, 95%CI: -62,-17)<br><br>Maternal risk genotype at rs1801133 predicted higher homocysteine concentration and lower birth weight (B= -61, 95%CI: -111, -10) (adjusted for gestational age, offspring gender and rs1801133 genotype)<br><br>Instrumental variable and triangulation analysis supported a causal association between maternal homocysteine concentration and offspring birth weight
7	Imbalance of folic acid and vitamin B12 is associated with birth outcome: an Indian pregnant women study<br><br>Gadgil et al., 2014 (22)	maternal plasma folate, vitamin B12 during pregnancy and their ratio	Observational study on 49 full-term pregnant women at a tertiary center in Pune. Concentrations of folate, B12 and homocysteine measured at 36 weeks gestation.<br><br>Neonatal anthropometry was measured within 24 h of birth	Neonatal anthropometry (birth weight, length, head circumference, abdominal circumference, mid arm circumference, chest circumference, triceps skinfold and subscapular skinfold thickness)	Maternal folate and B12 levels were not associated with neonatal anthropometry.<br><br>Folate to B12 ratio was negatively correlated with homocysteine, birth weight (p=0.009), length, head and chest circumference.

8	Association of homocysteine with global DNA methylation in vegetarian Indian pregnant women and neonatal birth anthropometrics<br><br>Gadgil et al., 2014 (70)	Plasma maternal folate, vitamin B-12 and homocysteine levels	49 participants having completed ≥36 weeks of pregnancy were enrolled in the study	Maternal global DNA methylation and neonatal anthropometrics (within 24 hours after birth)	No difference in global DNA methylation was found between the vegetarian and non-vegetarian pregnant women<br><br>Plasma total homocysteine of the vegetarian group showed significant correlation to global DNA methylation<br><br>Plasma total homocysteine was inversely related to tricep skinfold and chest circumference (r=-0.39, p=0.04) of neonates in vegetarian group
9	A prospective study of maternal fatty acids, micronutrients and homocysteine and their association with birth outcome<br><br>Wadhwani et al., 2015 (23)	Levels of maternal folate, vitamin B12 and homocysteine	106 women with normal pregnancy from tertiary hospital in Pune (total gestation >37 weeks and baby weight >2.5 kg and having no medical or obstetrical complications). Followed up every trimester starting from 16-20 weeks of gestation. Fasting blood samples obtained at every trimester	Birth outcome – Birth weight and chest circumference	Maternal plasma folate at first and third trimester and B12 at third trimester was positively associated (r=0.22) while maternal homocysteine at first trimester was negatively associated (r=-0.25) with birth weight
10	Trends of folate, vitamin B12 , and homocysteine levels	Maternal vitamin B_{12} , folate and	Prospective follow up of 100 women with	Adverse pregnancy outcomes (abortion, gestational diabetes,	Hyperhomocysteinemia in first trimester was associated with adverse pregnancy

	in different trimesters of pregnancy and pregnancy outcomes<br><br>Mishra et al., 2020 (42)	homocysteine levels during pregnancy	pregnancy without complications<br><br>Biochemical investigations (plasma homocysteine, folate, and vitamin B_{12} levels) were performed on all pregnant women in first, second, and third trimesters	hypertension, premature rupture of membranes) and low birth weight.	outcomes (OR = 3.62). Vitamin B12 deficiency in first trimester was associated with low birth weight babies (OR = 8.09)

Supplementary Table – 5 Indian studies on B12 during pregnancy and offspring biochemistry

Later life health outcomes in the offspring: Vitamin B12 status in offspring* (3 studies)					
**Sl. No.**	**Paper/ Author/ Year**	**Exposure**	**Test/Procedure**	**Outcomes**	**Results**
1	Child's homocysteine concentration at 2 years is influenced by pregnancy vitamin B12 and folate status<br><br>Lubree et al., 2012 (51)	Maternal B12, folate and homocysteine concentrations at 28 and 34 weeks pregnancy	118 rural and urban women attending a tertiary care center in Pune. Offspring measured at 2 years.	Offspring B12, foalte, homocysteine concentration at 2 years	Higher child vitamin B12 at 2y was associated with pregnancy vitamin B12 at 34 weeks (B=0.2, p=0.02 adjusted for current diet, breast feeding, cord B12 and folate)<br><br>Child's 2y tHcy concentration was associated with lower pregnancy vitamin B12 at 34 weeks (B=-0.2, p=0.02 adjusted for current diet, breast feeding, cord B12 and folate).
2	Contribution of Food Sources to the Vitamin B12 Status of South Indian Children from a Birth Cohort Recruited in the City of Mysore	Dietary intake of B12 rich foods in mothers during pregnancy and B12 concentrations at 28 week pregnancy	Children from the Mysore Parthenon Birth Cohort (n=512, 47.1% male)	Plasma B12 concentrations in the offspring at 9.5 years	Maternal pregnancy plasma B12 was associated with child's plasma B12 concentrations at 9.5 years (B=0.22, 95%CI

	Christian et al., 2015 (52)				0.14, 0.3 adjusted for current dietary intakes)
3	Vitamin B12 status in pregnant women and their infants in South India<br><br>Finkelstein et al 2017 (21)	Maternal B12 status at 14 weeks gestation and markers of B12 deficiency (MMA, homocysteine levels)	77 infants born to mothers who participated in an RCT and received 50mcg B12 or placebo starting from 14 weeks gestation	Infant B12 concentrations at 6 weeks	44% Infants were B12 deficient. Impaired B12 status (low maternal B12 and high MMA) in pregnancy was associated with higher infant B12 levels at 6 weeks (adjusted for B12 intervention). The strongest associations were associated with values in the third trimester ($p<0.01$). Infants born to women with vitamin B12 deficiency had a twofold greater risk of vitamin B12 deficiency ($P<0.01$). Higher maternal folate concentrations also predicted lower risk of vitamin B12 deficiency in infants ($P<0.05$).

Supplementary table -6 Indian studies on B12 during pregnancy and metabolic and cardiovascular outcomes

Later life health outcomes in the offspring: metabolic and cardiovascular outcomes* (5 studies)					
**Sl. No.**	**Paper/ Author/ Year**	**Exposure**	**Subjects/Procedure**	**Outcomes**	**Results**
1	Bone mass in Indian children--relationships to maternal nutritional status and diet during pregnancy: the Pune Maternal Nutrition Study<br><br>Ganpule et al., 2006 (53)	Vitamin B12, red cell folate and homocysteine measurements at 18 and 28 weeks pregnancy.	695 offspring from Pune Maternal Nutrition Study cohort and their parents	Total body and total spine bone mineral content and density (BMD) measure in offspring at 6 years of age by Dual energy Xray Absorptiometry (DXA)	Maternal red cell folate at 28 wks was associated with total (B=0.13, p<0.01) and spine (B=0.17p<0.001) bone mineral density, adjusted for parental size and body fat mass.
2	Vitamin B12 and folate concentrations during pregnancy and insulin resistance in the offspring: the Pune Maternal Nutrition Study<br><br>Yajnik CS et al., 2008 (19)	Maternal vitamin B12, folate and tHcy status = at 18 and 28 weeks of pregnancy	700 women pregnant women and their offspring from the Pune Maternal Nutrition Study Cohort<br><br>Offspring assessed at 6 years for anthropometry, body composition (dual-energy X-ray absorptiometry scan) and insulin resistance (homeostatic model assessment of insulin resistance [HOMA-R])	Offspring adiposity and insulin resistance at 6 years	Higher maternal erythrocyte folate concentrations at 28 weeks was associated with higher offspring adiposity and higher HOMA-R (B=0.38, p<0.001)<br>Low maternal vitamin B12 (18 weeks) predicted higher HOMA-R (B=-0.16, p=0.03) in the children<br><br>Offspring of mothers with a combination of high

					folate and low vitamin B12 concentrations were the most insulin resistant
3	Low maternal vitamin B12 status during pregnancy is associated with reduced heart rate variability indices in young children.<br><br>Sucharita et al., 2014 (56)	Mother's vitamin B12 levels during first trimester of pregnancy divided into groups higher and lower B12 based on median value.	79 healthy children whose mothers had been recruited during pregnancy as part of cohort studies from tertiary hospital in Bangalore.	Cardiac autonomic nervous activity in children using heart rate variability (HRV) indices, measured between 3 to 8 years of age.	53% reduction in the HRV measured in the low-frequency bands<br>Significant association between low -frequency and total power HRV with cord blood vitamin B12 levels ($\rho = 0.31$ and 0.30).
4	Association between maternal folate concentrations during pregnancy and insulin resistance in Indian children<br><br>Krishnaveni et al., 2014 (54)	Maternal homocysteine, Vitamin B12, and folate concentration in third trimester of pregnancy	654 women from Mysore Parthenon cohort study assessed at 30 weeks gestation and their offspring assessed at 5, 9.5 and 13.5 years.	Birth weight, current glucose and insulin resistance.<br>Analysis adjusted for GDM, maternal and child body size, child B12, folate status).	Maternal homocysteine concentrations were inversely associated with all neonatal anthropometric measurements, and positively associated with glucose concentrations in the children at 5 years (B=0.17, p=0.007)<br><br>Higher maternal folate concentrations were associated with higher HOMA-IR in the children at 9.5 age (B=0.1, 95%CI 0.01. 0.2, p=0.03). There were no associations with maternal B12.

5	Maternal B12, Folate and Homocysteine Concentrations and Offspring Cortisol and Cardiovascular Responses to Stress<br><br>Krishnaveni et al., 2020 (55)	Maternal B12, folate, homocysteine at 30 wk pregnancy	264 adolescents from the Mysore Parthenon cohort study assessed at 13.6 years of age on the Trier Social Stress Test for Children<br>Baseline and poststress salivary cortisol concentrations, heart rate, blood pressure, stroke volume, cardiac output, and total peripheral resistance were measured continuously at baseline, during TSST-C, and for 10 minutes after the TSST-C	Offspring cortisol and cardiovascular responses to stress test	Maternal low B12 status (plasma B12 < 150 pmol/L) was associated with greater cortisol responses to stress in the offspring (B=0.36, 95%CI 0.16, 0.57). Higher homocysteine concentrations were associated with greater offspring heart rate response (B=0.13, p-0.01).

Supplementary Table – 7 Indian studies on B12 during pregnancy and offspring cognitive outcomes

Later life health outcomes in the offspring: cognitive outcomes (3 studies)					
**Sl. No.**	**Paper/ Author/ Year**	**Exposure**	**Test/Procedure**	**Outcomes**	**Results**
1	Vitamin B12 status of pregnant Indian women and cognitive function in their 9-year-old children<br><br>Bhate et al., 2008 (58)	Maternal plasma vitamin B12 status during pregnancy	Cognitive scores in offspring were compared between two groups chosen based on maternal B12 status during pregnancy (highest and lowest decile of B12 levels) from the PMNS cohort (n=108). Cognitive test included Coloured Progressive Matrices, Colour trails (A&B), Visual recognition test, Digit Span	Child's cognitive function at 9 years of age	Offspring of mothers in lowest decile of B12 had poorer performance on Digit span (Attention) and Color trails A test (executive function)
2	Higher Maternal Plasma Folate but Not Vitamin B-12 Concentrations during Pregnancy Are Associated with Better Cognitive Function Scores in 9- to 10- Year-Old Children in South India<br><br>Veena et al., 2010 (59)	Maternal folate, vitamin B-12 and homocysteine during 30 weeks pregnancy	536 children (aged 9–10 y) from the Mysore Parthenon birth cohort Outcome measured on the Kaufman Assessment Battery	Offspring cognitive function at 9.5 years of age.	Children's cognitive test scores increased by 0.1–0.2 per SD increase across the entire range of maternal folate concentrations (adjusted for gender, age of child, socio-economic factors and child's current folate, B12 levels)

					There were no associations of maternal B12 and homocysteine with offspring cognitive scores.
3	Vitamin B12 and folate during pregnancy and offspring motor, mental and social development at 2 years of age<br><br>Bhate et al., 2012 (57)	Maternal B12 and folate status during pregnancy at 28 and 34 weeks gestation	Mothers (n = 123) and their offspring (62 girls, 61 boys) from rural and middle-class urban communities in and around Pune city were followed through pregnancy up to 2 y Outcomes assessed on the Developmental Assessment Scale for Indian Infants and the Vineland Social Maturity Scale	Offspring motor, mental and social development at two years of age (2 y)	Motor development at 2yrs was associated with maternal folate during pregnancy (r=0.2).<br><br>Mental and Social development quotient were positively associated with maternal B12 and 28 weeks (B=0.27) and folate at 28 and 34 weeks (B=0.2)<br><br>All associations were independent of child's B12 and folate status at 2 years.

Supplementary table – 8 Studies from India on supplementation of B12 and/or multiple micronutrients or micronutrient rich food during pregnancy and maternal and child outcomes

Supplementation studies (7 studies)					
**Sl. No.**	**Paper/ Author/ Year**	**Subjects and timing of intervention**	**Dose and type of intervention**	**Outcomes**	**Results**
1	Multi micronutrient supplementation for undernourished pregnant women and the birth size of their offspring: a double-blind, randomized, placebo-controlled trial<br><br>Gupta et al., 2007 (40)	200 pregnant undernourished women (BMI<18.5kg/m2 and/or Hb 7 to 9 gm%) in their second trimester from tertiary hospital in New Delhi	Randomized to receive micronutrient supplementation (29 vitamins and minerals including 1mcg B12) or placebo from second trimester till delivery.	Birth size of their offspring, incidence of low-birth-weight infants and early neonatal morbidity	Infants in the micronutrient group were heavier by 98 g (adjusted p =0.09) and measured 0.80 (p=0.04) cm longer and 0.20 cm (p=0.02) larger in midarm circumference compared with the placebo group<br><br>Incidence of LBW declined from 43.1% to 16.2% (adjusted RR=0.3, 95%CI 0.13,0.71) with multi micronutrient supplementation and early neonatal morbidity declined from 28.0% to 14.8% (RR=0.42, 95%CI 0.19, 0.94)

2	Vitamin B-12 Supplementation during Pregnancy and Early Lactation Increases Maternal, Breast Milk, and Infant Measures of Vitamin B-12 Status<br><br>Duggan et al., 2014 (47)	Intervention in pregnancy from first trimester (<14 weeks gestation) till 6 weeks postpartum in women from lower SES in urban Bangalore	Randomized, placebo-controlled clinical trial. (n=183 in each group)<br><br>50mcg oral B12 or placebo. Both groups received Iron-Folic acid supplements	Maternal, breast milk and infant concentrations of vitamin B-12	Compared with placebo vitamin B-12–supplemented women had significantly higher plasma vitamin B-12 concentrations at both 2nd & 3rd trimesters and at 6 weeks postpartum (136 vs 87pM).<br><br>In a subset of infants tested at 6 weeks of age, in the supplemented group, median plasma vitamin B-12 concentration was higher (199 vs 139pM), plasma methylmalonic acid and Hcy concentrations were significantly lower<br>No difference in birth weight between the groups (2.85 6 0.46 kg in the vitamin B-12 group vs. 2.83 6 0.45 kg in the placebo group)
3	Improving women's diet quality pre-conceptionally and during gestation:	Preconceptional intervention	Non-blinded, individually randomized controlled trial	Birth weight (LBW)<br>Secondary birth outcomes were	There was no overall increase in birth weight

	effects on birth weight and prevalence of low birth weight—a randomized controlled efficacy trial in India (Mumbai Maternal Nutrition Project)<br><br>Potdar et al., 2014 (45)	Total randomized women 6512, live births – 1962, outcome measurements on – 1360 newborn. Subjects from Mumbai urban slums<br><br>Intervention started more than 90 days before pregnancy till delivery.	Intervention group received daily snack – 'samosa' made from green leafy vegetables, fruit, and milk. Control group received a daily low-micronutrient vegetable snack (potato and onion)	gestational age, small for gestational age, other new born anthropometry measurements	in the intervention group.<br>In new-borns whose mothers started supplementation >90 days before pregnancy, birth weight was higher in the treatment group by 48gms.<br>There was a significant interaction with maternal pre-pregnancy BMI. Mothers in supplementation group with higher pre pregnancy BMI had higher birth weight.
4	A Daily Snack Containing Leafy Green Vegetables, Fruit, and Milk before and during Pregnancy Prevents Gestational Diabetes in a Randomized, Controlled Trial in Mumbai, India<br><br>Sahariah et al., 2016 (33)	Preconceptional intervention<br><br>Total randomized women 6512, live births – 1962, outcome measurements on – 1360 newborn. Subjects from Mumbai urban slums<br><br>Intervention started more than 90 days before pregnancy till delivery.	Non-blinded, individually randomized controlled trial<br><br>Intervention group received daily snack – 'samosa' made from green leafy vegetables, fruit, and milk. Control group received a daily low-micronutrient vegetable snack (potato and onion)<br><br>Outcome assessment (Oral glucose tolerance test at 28	Risk of Gestational diabetes (GDM)	Prevalence of GDM was reduced in the treatment group (7.3% compared with 12.4% in controls

			wk) available in 1008 women.		
5	Effect of a micronutrient-rich snack taken pre conceptionally and throughout pregnancy on ultrasound measures of fetal growth: The Mumbai Maternal Nutrition Project (MMNP)<br><br>Lawande et al., 2018 (46)	Preconceptional intervention<br><br>Total randomized women 6512, live births – 1962, outcome measurements on – 1360 newborn. Subjects from Mumbai urban slums<br><br>Intervention started more than 90 days before pregnancy till delivery.	Non-blinded, individually randomized controlled trial<br><br>Intervention group received daily snack – 'samosa' made from green leafy vegetables, fruit, and milk. Control group received a daily low-micronutrient vegetable snack (potato and onion)<br><br>Outcome measurements: Fetal ultrasound(n=1677) 1st trimester: foetal crown-rump length (9-12 weeks) Measurements in 2nd and 3rd trimester: head circumference, biparietal diameter, femur length, and abdominal circumference (19-20 weeks; 28-30 weeks)	Ultrasound measures of fetal size	Intervention had no effect on fetal size or growth at any stage of pregnancy
6	Effects of maternal vitamin B12 supplementation on early infant neurocognitive outcomes: a randomized controlled clinical trial.	Maternal B12 supplementation status and vitamin B12, red cell folate and homocysteine concentrations at 12, 28 and 33 weeks gestation.	Randomized, placebo-controlled clinical trial of oral B12 supplementation (50 µg) beginning at <14 weeks of gestation till 6-week post-partum in 183	Cognitive development in infants at 9 months of age on domains of cognitive, language and motor development.	No difference in cognitive outcomes between intervention groups.<br>Higher Maternal homocysteine during pregnancy was

	Srinivasan et al., 2017 (61)		pregnant women in each group<br><br>Outcome measurements in 178 offspring		associated with poorer expressive language and fine motor functions at 9 months of age. (strongest association for homocysteine in second trimester with expressive language, B=-3.13 adjusted for birth weight, treatment group, income, home environment, parity)
	Effect of Maternal Vitamin B12 Supplementation on Cognitive Outcomes in South Indian Children: A Randomized Controlled Clinical Trial<br><br>Thomas et al., 2019 (60)	Maternal B12 supplementation status and vitamin B12, red cell folate and homocysteine concentrations at 12, 28 and 33 weeks gestation.	Randomized, placebo-controlled clinical trial of oral B12 supplementation (50 µg) beginning at <14 weeks of gestation till 6-week post-partum in 183 pregnant women in each group<br><br>Outcome measurements in 218 offspring	Cognitive development in infants at 30 months of age on domains of cognitive, language and motor development.	Children of mothers who received oral vitamin B12 supplementation had significantly higher scores on expressive language (B=0.14)<br><br>Maternal homocysteine in second and third trimester continued to be associated negatively with expressive language scores even at 30 months (B=-0.18)

Supplementary Table 9 – Gene polymorphisms and associations with outcomes

Gene	Outcome	Findings
MTHFR<br><br>Puri et al., 2013 (27)	Recurrent pregnancy loss	MTHFR genotypic distribution among cases and controls showed no significant difference<br><br>MTHFR C677T polymorphism was found to be significantly associated with increased homocysteine in the case group
MTHFR<br>Nair 2012 (68)	Recurrent pregnancy loss	Homozygosity and heterozygosity for the MTHFR C677T polymorphism confer a 6.3- and 1.9-fold increased risk of idiopathic REPL, respectively.
MTHFR<br><br>Kaur et al., 2018 (28)	Recurrent pregnancy loss	MTHFR 677T allele frequency was highest in non-anemic RPL women, who exhibited highest homocysteine levels with the highest B12/folate ratios. A higher allelic frequency for C677T allele was observed (0.16 versus 0.11) in the case group
MTHFR<br>Folate Hydrolase (GCP II)<br><br>Naushad & Devi, 2010 (69)	NTD	Maternal MTHFR C677T (OR=2.69) and parental GCP II C1561T polymorphisms (OR = 3.23) are associated with increased risk for NTDs<br><br>Both paternal and maternal GCP II T-variant alleles were found to interact with MTHFR 677T- and MTRR G-variant alleles in increasing the risk for NTD
MTHFR<br><br>Yajnik CS et al., 2014 (44)	Offspring birth weight	Maternal risk genotype at rs1801133 predicted higher homocysteine concentration and lower birth weight (adjusted for gestational age, offspring gender and rs1801133 genotype)
MTHFR gene polymorphism<br><br>Deb et al., 2011 (35)	NTD	Muslim NTD mothers had higher TT genotype showing increased risk for neural tube defects (OR = 12.9 adjusted for mothers age, education economic status)

MTHFR<br>TCN2<br><br>Godbole et al., 2011 (37)	NTD	1298A>C and 1781G>A polymorphisms in MTHFR were protective<br><br>Maternal 776C>G polymorphism in TCN2 was strongly predictive of NTD in the offspring
